# Exosome-nanomaterial hybrid nanomedicine for ischemic stroke: microenvironment-informed design, therapeutic applications, and translational challenges

**DOI:** 10.3389/fncel.2026.1835402

**Published:** 2026-06-22

**Authors:** Qi Zhu, Xiaodan Wu

**Affiliations:** 1 Department of Emergency, Central Hospital Affiliated to Shenyang Medical College, Shenyang, China; 2 Department of Traditional Chinese Medicine, Central Hospital Affiliated to Shenyang Medical College, Shenyang, China

**Keywords:** blood–brain barrier, exosomes, extracellular vesicles, hybrid nanoparticles, ischemic stroke, nanomedicine, neurovascular repair, thromboinflammation

## Abstract

Ischemic stroke (IS) remains a major cause of mortality and long-term disability despite advances in reperfusion therapy, underscoring the need for adjunctive interventions that can operate within the dynamic post-ischemic microenvironment. Exosomes and other extracellular vesicles (EVs) provide a biologically compatible interface for brain delivery, yet native vesicles are constrained by heterogeneous composition, modest loading efficiency, limited targeting control, and manufacturing variability. Exosome-nanomaterial hybrid systems are therefore emerging as modular platforms that integrate exosomal biointerfaces with the tunable payload capacity, imaging compatibility, mechanical stability, and stimulus responsiveness of synthetic nanomaterials. In this review, we propose a microenvironment-informed design paradigm for IS nanomedicine. In this framework, the ischemic lesion is not treated as a passive delivery destination, but as a staged design brief defined by blood–brain barrier (BBB) remodeling, thromboinflammation, oxidative stress, immune-cell trafficking, and neurovascular repair. We summarize how exosome source, nanomaterial component, cargo loading, surface functionalization, administration route, and characterization strategy can be selected according to these pathological cues. We further discuss therapeutic applications in BBB-penetrant delivery, neuroprotection, inflammatory modulation, imaging-guided therapy, and neurovascular recovery, together with safety, quality-control, manufacturing, and regulatory barriers. Overall, exosome-nanomaterial hybrids may become clinically meaningful for IS only when their design is microenvironment-informed, mechanism-driven, and translationally scalable.

## Graphical abstract

**Figure:**
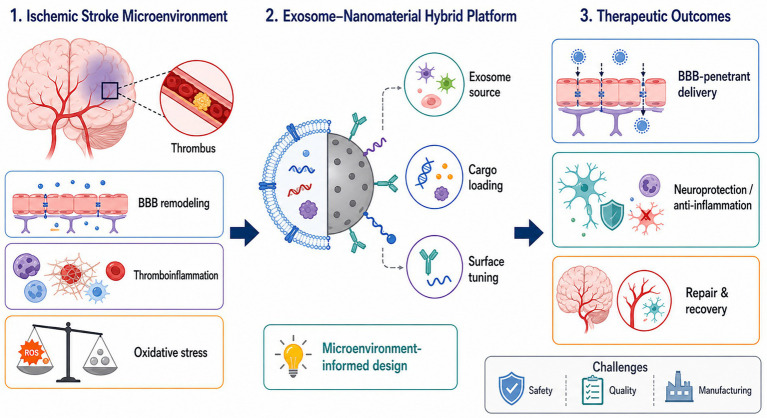
Microenvironment-informed design of exosome-nanomaterial hybrid nanomedicine for ischemic stroke. The ischemic microenvironment provides staged pathological cues, which are translated into modular selection of exosome source, nanomaterial component, cargo, targeting strategy, and validation endpoint.

## 1 Introduction

Ischemic stroke (IS) remains a leading cause of death and long-term disability worldwide, and its global burden is still substantial despite major advances in acute reperfusion therapy (Li X. Y. et al., 2024). Intravenous thrombolysis and endovascular thrombectomy have improved outcomes in selected patients, yet many patients remain ineligible for timely recanalization, and poor functional recovery is still common even after technically successful reperfusion (Chen et al., 2024). This persistent gap reflects the complex pathobiology of IS, in which excitotoxicity, oxidative stress, blood–brain barrier (BBB) disruption, thromboinflammation, microvascular dysfunction, and delayed neurovascular remodeling evolve in a spatially and temporally heterogeneous manner (Hirsch et al., 2023). Consequently, effective post-stroke intervention requires not only potent therapeutic agents, but also delivery systems capable of navigating the BBB, localizing to injured tissue, and adapting to stage-specific pathological cues.

Extracellular vesicles (EVs), especially exosomes and small EVs, have emerged as attractive candidates in this setting because they combine endogenous biological communication with nanoscale carrier properties (Hirsch et al., 2023). As naturally derived lipid-bilayer vesicles, they exhibit favorable biocompatibility, relatively low immunogenicity, and a capacity to transport proteins, lipids, and nucleic acids. In experimental stroke, native and engineered EVs have been reported to attenuate neuroinflammation, reduce apoptosis, support angiogenesis and neurogenesis, and improve neurological recovery; importantly, recent preclinical evidence suggests that engineered EVs may outperform unmodified vesicles, indicating that therapeutic benefit depends not only on vesicle origin, but also on rational manipulation of cargo, surface properties, and targeting behavior (Li P. et al., 2023; Chen et al., 2025).

At the same time, native EVs remain constrained by limited loading capacity, heterogeneous composition, incomplete control over biodistribution, and challenges in scale-up and quality consistency (Chen et al., 2025). These limitations have driven increasing interest in exosome-nanomaterial hybrid systems, which aim to combine the biological advantages of exosomes with the tunable physicochemical functions of synthetic nanomaterials. In principle, hybridization can improve structural stability, payload capacity, lesion targeting, imaging compatibility, and stimulus-responsive release while retaining the membrane-mediated compatibility that makes exosomes attractive for brain delivery. The rationale for focusing on exosome-enriched small EVs rather than broader EV populations is that their size range, endosomal origin, established marker framework, and extensive stroke literature make them a practical starting point for controlled hybrid engineering, whereas other EV subtypes may require different isolation, characterization, and quality-control strategies. Recent quality and safety guidance also emphasizes that EV therapeutics must be evaluated through linked production, purification, characterization, quality evaluation, safety assessment, and clinical-development workflows (Takakura et al., 2024). In this review, we discuss exosome-nanomaterial hybrid nanomedicine for IS from a design-oriented perspective, moving from the pathophysiological basis of the ischemic microenvironment to platform engineering, therapeutic and theranostic applications, and the principal challenges that will shape future development.

### 1.1 Toward a microenvironment-informed design paradigm

The central thesis of this review is that the ischemic microenvironment should be interpreted not merely as a therapeutic target, but as a design brief for next-generation nanomedicines. In this paradigm, each dominant pathological feature of IS is translated into a specific engineering question. BBB remodeling raises questions of transport route, endothelial interaction, and barrier preservation; thromboinflammation raises questions of vascular targeting, platelet–neutrophil interaction, and neutrophil extracellular trap (NET)-associated release cues; oxidative stress raises questions of antioxidant capacity and reactive oxygen species (ROS)-responsive disassembly; and recovery-phase remodeling raises questions of sustained retention, trophic support, and neurovascular repair.

Accordingly, the proposed feedback loop should link the pathophysiological cue to a rational design principle, followed by modular engineering and functional validation. A lesion cue such as P-selectin expression, excessive ROS, endothelial activation, protease activity, microglial polarization, or BBB transport reprogramming should first define the desired carrier behavior. The exosome source, nanomaterial core, cargo class, surface ligand, and administration route should then be selected to execute that behavior. Finally, validation should confirm that the engineered platform responds to the intended cue and produces measurable therapeutic benefit in disease-relevant models.

## 2 Pathophysiological and design basis

### 2.1 Blood–brain barrier dysfunction and brain delivery barriers

The BBB should no longer be viewed as a passive wall that simply ruptures after arterial occlusion; rather, it is a dynamically remodeled neurovascular interface whose permeability evolves across ischemia and reperfusion (Zhu et al., 2022; Sugiyama et al., 2023; Qin et al., 2023; Zhang X. L. et al., 2024). Endothelial glycocalyx loss is an early determinant of BBB failure, because glycocalyx degradation increases tracer leakage and brain edema by promoting caveolin-1-dependent endothelial transcytosis even before generalized structural collapse of tight junctions becomes evident (Zhu et al., 2022). Consistently, occludin deficiency aggravates BBB permeability and neurological dysfunction from the acute to the chronic phase after experimental ischemic stroke, underscoring that junctional destabilization remains a sustained determinant of barrier failure rather than a purely transient event (Sugiyama et al., 2023). Endothelial stress signaling also critically shapes barrier integrity, as endothelial overexpression of mitogen-activated protein kinase phosphatase-1 reduces inflammatory activation, preserves occludin expression, and alleviates BBB injury after transient middle cerebral artery occlusion (tMCAO) (Qin et al., 2023).

Recent studies further indicate that post-ischemic BBB dysfunction cannot be explained by paracellular leakage alone, because transcellular transport is also actively reprogrammed after stroke (Zhu et al., 2022; Zhang X. L. et al., 2024; Hu et al., 2024). Glial growth factor 2 alleviates ischemia/reperfusion-compromised BBB integrity by suppressing both Mfsd2a/Cav-1-mediated transcellular permeability and Pdlim5/YAP/TAZ-associated paracellular injury, directly linking barrier dysfunction to coordinated changes in vesicular transport and endothelial cytoskeletal signaling (Zhang X. L. et al., 2024). Likewise, nicotine-mediated regulation of endothelial Pdlim5 attenuates BBB damage after acute ischemic stroke, further highlighting the importance of endothelial scaffolding programs in post-ischemic barrier remodeling (Hu et al., 2024). Multi-omics-guided work has additionally identified Slc22a8 as an endothelial/pericyte-associated determinant of BBB integrity whose restoration enhances tight-junction protein expression and reduces BBB leakage after cerebral ischemia/reperfusion (Liu et al., 2025). Endothelial LRRC8A has also been implicated as a pathogenic mediator of aberrant BBB permeability in ischemic stroke, with its inhibition promoting ZO-1 and VE-cadherin preservation and improving stroke outcome (Tian et al., 2025).

BBB dysfunction in ischemic stroke is further amplified by inflammatory, matrix-degrading, and ferroptotic signals (Deng et al., 2023; Liu et al., 2025). Upregulation of miR-671-5p attenuates BBB disruption in middle cerebral artery occlusion/reperfusion (MCAO/R) models by restraining nuclear factor κB (NF-κB)/matrix metalloproteinase 9 (MMP-9) signaling and preserving claudin-5, occludin, and ZO-1, indicating that barrier breakdown is tightly coupled to inflammatory transcriptional programs (Deng et al., 2023). In parallel, macrophage-derived exosomal thrombospondin 1 (THBS1) induces endothelial ferroptosis and aggravates BBB disruption during cerebral ischemia/reperfusion injury, demonstrating that extracellular vesicle cargo can actively worsen vascular injury rather than simply reflect it (Liu et al., 2025). Collectively, these studies support the view that the ischemic BBB is a transport-reprogrammed and inflammation-sensitive interface rather than a uniformly “open” barrier.

For nanomedicine design, this evolving view of BBB biology has two direct implications (Zhu et al., 2022; Zhang X. L. et al., 2024; Liu et al., 2025). First, the post-stroke BBB is spatially heterogeneous and temporally phased, so passive leakage alone is unlikely to guarantee efficient or reproducible brain delivery (Zhu et al., 2022; Sugiyama et al., 2023; Zhang X. L. et al., 2024). Second, delivery systems that can engage receptor-mediated transport, adapt to endothelial stress states, or even stabilize barrier function are more rational than systems that merely exploit pathological leakiness (Zhang X. L. et al., 2024; Hu et al., 2024; Liu et al., 2025; Liu et al., 2025). In other words, successful stroke nanomedicine should be built not only to cross the compromised BBB, but also to operate within its dynamic biology.

These observations indicate that physiological and pathological BBB states should be considered as distinct design contexts for hybrid nanomedicine. Under physiological conditions, a clinically acceptable system should preserve tight junctions, minimize endothelial toxicity, and avoid unnecessary barrier opening. In the ischemic brain, transient permeability, receptor-mediated transport, endothelial stress signaling, glycocalyx loss, and inflammatory activation may provide opportunities for delivery. These opportunities should be used cautiously because excessive barrier disruption may increase edema, hemorrhagic transformation, or off-target inflammatory injury.

### 2.2 Thromboinflammation and neurovascular injury

Thromboinflammation has emerged as a central organizing principle in ischemic stroke, replacing the older view that thrombosis and inflammation are largely sequential processes (Lopez-Pedrera et al., 2023; Jabrah et al., 2024; Lapostolle et al., 2023; Denorme et al., 2022). Proteomic analysis of thrombectomy-retrieved thrombi demonstrates a prominent innate immune and neutrophil-related signature associated with stroke etiology, severity, and prognosis, indicating that the occluding clot is an immunologically active structure rather than a passive mechanical plug (Lopez-Pedrera et al., 2023). Histopathologic analyses of acute ischemic stroke clots further show that white blood cell subtypes and neutrophil extracellular traps (NETs) vary across stroke etiologies, with cardioembolic thrombi showing higher neutrophil and peripheral NET enrichment (Jabrah et al., 2024). Clinically, NET-rich thrombi are associated with poorer recanalization and worse outcome, supporting the concept that thromboinflammation is embedded within clot architecture itself (Lapostolle et al., 2023).

Mechanistically, recent studies position the endothelial–platelet–neutrophil axis at the center of acute stroke thromboinflammation (Denorme et al., 2022; Dhanesha et al., 2022; Pena-Martinez et al., 2022; De Wilde et al., 2023). Denorme et al. showed that NET-forming neutrophils accumulate throughout ischemic brain tissue and that platelet-derived high mobility group box 1 (HMGB1) is a key trigger of acute post-stroke NETosis, whereas platelet depletion or platelet-specific HMGB1 deletion reduces NET burden and improves outcome (Denorme et al., 2022). Dhanesha et al. further demonstrated that neutrophil pyruvate kinase M2 (PKM2) is upregulated after stroke and drives neutrophil hyperactivation and NET formation, thereby linking thromboinflammation to metabolic reprogramming within myeloid cells (Dhanesha et al., 2022). In a fibrin-rich thrombotic stroke model, pharmacologic NET targeting remained neuroprotective despite limited reperfusion benefit, suggesting that NETs contribute not only to thrombus structure but also to downstream tissue injury (Pena-Martinez et al., 2022). Spatial analysis of murine ischemia/reperfusion stroke additionally indicates that NET formation follows a spatiotemporally regulated pattern and may cooperate with von Willebrand factor to sustain thromboinflammatory damage (De Wilde et al., 2023).

This spatial organization deserves explicit distinction because NETs located in different anatomical compartments are likely to contribute to different phases of post-ischemic injury. Intravascular NETs are preferentially detected within ischemic brain vessels during early ischemia/reperfusion and are mechanistically aligned with von Willebrand factor-associated secondary microthrombosis and impaired microvascular reperfusion (De Wilde et al., 2023). Perivascular neutrophil accumulation, together with occasional NET formation along this trafficking route, is most relevant to the neurovascular interface: after cerebral ischemia/reperfusion, neutrophils accumulate in perivascular spaces with basement-membrane disruption, supporting the interpretation that this compartment links vascular inflammation to blood–brain barrier injury rather than merely reflecting intraluminal thrombosis (Otxoa-de-Amezaga et al., 2019). Parenchymal NETs have been demonstrated in the peri-infarct cortex and other ischemic brain regions in several models, where they can persist beyond the hyperacute phase and are associated with delayed vascular remodeling defects and local tissue injury, although the extent of parenchymal NET accumulation varies across experimental settings (Kang et al., 2020; Li C. et al., 2023). Accordingly, intravascular NETs are most directly relevant to thrombus stabilization and microvascular no-reflow, perivascular NETs to blood–brain barrier and neurovascular-unit destabilization, and parenchymal NETs to sustained local inflammatory injury and impaired repair. For hybrid nanomedicine, this compartmental distinction is not merely descriptive: vascular-facing NET-directed carriers may be better suited to intravascular secondary thrombosis, blood–brain barrier-stabilizing or perivascular-retentive systems to neurovascular-interface injury, and deeper brain-penetrant formulations to parenchymal NET-associated tissue damage.

The thromboinflammatory response is also biologically heterogeneous and incompletely resolved over time (Datsi et al., 2022; Li J. et al., 2024; Patel et al., 2023). Stroke-derived neutrophils display enhanced NET-forming capacity and impaired DNase-I-mediated NET resolution compared with neutrophils from healthy controls, implying that post-stroke neutrophils are functionally reprogrammed rather than merely numerically increased (Datsi et al., 2022). In patients with large artery atherosclerotic stroke, circulating myeloperoxidase-DNA complexes (MPO-DNA), peptidylarginine deiminase 4 (PAD4), complement component 1q (C1q), interleukin (IL)-1β, IL-6, and IL-8 rise early after stroke onset, and baseline MPO-DNA, absent in melanoma 2 (AIM2), and IL-1β correlate with poor 3-month outcome and later vascular events (Li J. et al., 2024). These findings indicate that thromboinflammation in ischemic stroke is a staged process involving persistent NETosis, inflammasome-related signaling, and incomplete resolution (De Wilde et al., 2023; Datsi et al., 2022; Li J. et al., 2024).

The neurovascular consequences of thromboinflammation extend well beyond the primary occluding clot (Patel et al., 2023; Huang et al., 2022; Dai et al., 2025). In obese hyperglycemic mice, neutrophil-specific deletion or pharmacologic inhibition of integrin α9 reduces post-ischemic thrombosis, intracerebral fibrin(ogen) and platelet deposition, inflammatory cytokines, and circulating NET markers while improving cerebral blood flow and long-term neurological outcome (Patel et al., 2023). Edaravone dexborneol also suppresses NET formation in both acute ischemic stroke patients and middle cerebral artery occlusion (MCAO) mice, and its neurovascular protection is accompanied by improved tight-junction-associated protein expression and reduced BBB permeability (Huang et al., 2022). Single-cell and validation studies further show that endothelial cells dynamically regulate immune-cell infiltration after stroke, with intercellular adhesion molecule 1 (ICAM-1)-high venous endothelial cells predominating in the acute phase and vascular cell adhesion molecule 1 (VCAM-1)-high venous endothelial cells becoming more prominent later, thereby reshaping the cellular composition of the post-stroke inflammatory niche (Dai et al., 2025). Together, these studies suggest that thromboinflammation is both a determinant of vascular occlusion and a driver of secondary neurovascular injury (Lopez-Pedrera et al., 2023; Jabrah et al., 2024; Lapostolle et al., 2023; Denorme et al., 2022; Dhanesha et al., 2022; Pena-Martinez et al., 2022; De Wilde et al., 2023; Datsi et al., 2022; Li J. et al., 2024; Patel et al., 2023; Huang et al., 2022; Dai et al., 2025).

The thromboinflammatory response also has direct implications for platform design. When the therapeutic goal is acute-phase intervention, vascular-facing and immune-cell-responsive modules may be more appropriate than broadly distributed carriers. Relevant design features include platelet- or endothelial-interacting membranes, ligands that recognize activated platelets or inflamed endothelium, responsive linkers sensitive to ROS, shear stress, elastase, or other proteases, and cargoes that suppress NETosis, inflammasome activation, or endothelial injury. Functional validation should therefore include assays that capture platelet–neutrophil aggregation, NET formation, endothelial adhesion, microvascular obstruction, and downstream neurological outcome.

### 2.3 Implications of the ischemic microenvironment for nanomedicine design

The ischemic microenvironment is spatially heterogeneous, temporally evolving, and biologically multiplex, and these features should directly inform nanomedicine design (Zhu et al., 2022; Zhang X. L. et al., 2024; Lopez-Pedrera et al., 2023; Denorme et al., 2022; De Wilde et al., 2023; Dai et al., 2025). In practice, this means that stroke nanomedicines should be pathology-responsive rather than constitutively active, because the relevant cues in the ischemic core, penumbra, intravascular thrombus, and reperfused microvasculature are not identical (Zhu et al., 2022; Zhang X. L. et al., 2024; Lopez-Pedrera et al., 2023; Denorme et al., 2022). The design objective is therefore not simply to “reach the brain”, but to match carrier behavior to lesion-stage biology, including oxidative stress, endothelial activation, thrombosis-associated shear conditions, innate immune cell trafficking, and barrier remodeling (Zhu et al., 2022; Zhang X. L. et al., 2024; Denorme et al., 2022; Dai et al., 2025).

Among these cues, oxidative stress remains one of the most actionable triggers for lesion-adaptive nanomedicine (Mu et al., 2023; Zhao et al., 2022; Cao et al., 2024; Nagareddy et al., 2024; Jian et al., 2025; Chen et al., 2025). Mu et al. designed neutrophil-hitchhiking ligustrazine nanoparticles in which a formyl peptide receptor (FPR)-binding surface motif enabled neutrophil-assisted accumulation at inflamed ischemic sites, while ROS-responsive bond cleavage triggered local drug release after delivery (Mu et al., 2023). Zhao et al. developed a transferrin-enabled manganese-based nanozyme that combined BBB transport, ROS scavenging, and weak-acid-activated magnetic resonance imaging (MRI) capability, illustrating how the oxidative and acidic lesion milieu can be integrated into both therapy and imaging (Zhao et al., 2022). In another study, a sulfated polysaccharide-based nanocarrier with ROS-responsive disassembly and a stroke-homing peptide promoted microglial polarization, preserved BBB integrity, and improved neurovascular remodeling in tMCAO mice (Cao et al., 2024). Similar logic underlies atorvastatin-loaded chitosan–bilirubin nanoparticles, quercetin-based polydopamine nanoparticles, and BBB-penetrant metal–organic framework antioxidant nanozymes, all of which used elevated ROS as a release cue or catalytic target to improve delivery efficiency and broaden neuroprotection in ischemic stroke models (Nagareddy et al., 2024; Jian et al., 2025; Chen et al., 2025).

The thromboinflammatory and vascular microenvironment also provides highly specific targeting opportunities that are especially relevant for hybrid and biomimetic platforms (Denorme et al., 2022; Dhanesha et al., 2022; Pena-Martinez et al., 2022; De Wilde et al., 2023; Datsi et al., 2022; Li J. et al., 2024; Patel et al., 2023; Huang et al., 2022; Dai et al., 2025; Kong et al., 2024; Zhang H. et al., 2025; Tang et al., 2024; Wang et al., 2025; Li X. et al., 2025). Kong et al. engineered an ultrasmall Cu/Cu_2_O nanoparticle-based diselenide-bridged nanoplatform that combined ROS scavenging with neuronal membrane enhancement for targeted ischemic stroke therapy, thereby linking redox-responsive material design to downstream neuroprotection (Kong et al., 2024). Zhang et al. subsequently designed a shear force–ROS sequential responsive system in which fucoidan targeted P-selectin on activated platelets, elevated shear force triggered early thrombolytic release, and subsequent ROS exposure promoted neuroprotective drug release in the ischemic lesion (Zhang H. et al., 2025). Tang et al. further showed that an elastase-targeting biomimetic nanoplatform coated with platelet–neutrophil hybrid membranes could suppress NETosis, inhibit AIM2 inflammasome activation, and promote neurovascular remodeling, demonstrating the value of designing carriers around neutrophil elastase and NET-associated pathology (Tang et al., 2024). Platelet membrane-based deferoxamine liposomes and other multifunctional biomimetic nanoplatforms provide additional evidence that lesion-associated vascular damage, ferroptosis, complement activation, and inflammatory endothelium can be converted from therapeutic barriers into targeting handles (Wang et al., 2025; Li X. et al., 2025).

These studies collectively suggest that the optimal stroke nanoplatform is unlikely to be a single-cue carrier (Mu et al., 2023; Zhao et al., 2022; Cao et al., 2024; Nagareddy et al., 2024; Jian et al., 2025; Chen et al., 2025; Kong et al., 2024; Zhang H. et al., 2025; Tang et al., 2024; Wang et al., 2025; Li X. et al., 2025). Instead, design is moving toward multi-stage and multi-cue systems that combine BBB penetration, thromboinflammatory targeting, and lesion-responsive payload release within one integrated architecture (Zhao et al., 2022; Cao et al., 2024; Kong et al., 2024; Zhang H. et al., 2025; Tang et al., 2024; Wang et al., 2025; Li X. et al., 2025). This logic is particularly relevant to exosome–nanomaterial hybrids, because exosomes can contribute biological membrane compatibility, BBB permeability, and intrinsic immunomodulatory activity, whereas synthetic nanomaterials can add structural stability, scalable loading, and triggerable release functions (Liu et al., 2025; Xie et al., 2025). A recent exosome–liposome hybrid study in MCAO/R mice exemplifies this direction, showing that fusion of neural stem cell-derived exosomes with liposomes enabled simultaneous modulation of neuroinflammation and lipid metabolism while improving functional recovery (Xie et al., 2025). Taken together, the ischemic microenvironment argues for nanomedicine platforms that are stage-matched, biomimetic, and mechanistically coupled to BBB remodeling and thromboinflammatory reprogramming rather than relying on nonspecific accumulation alone.

**Figure 1:**
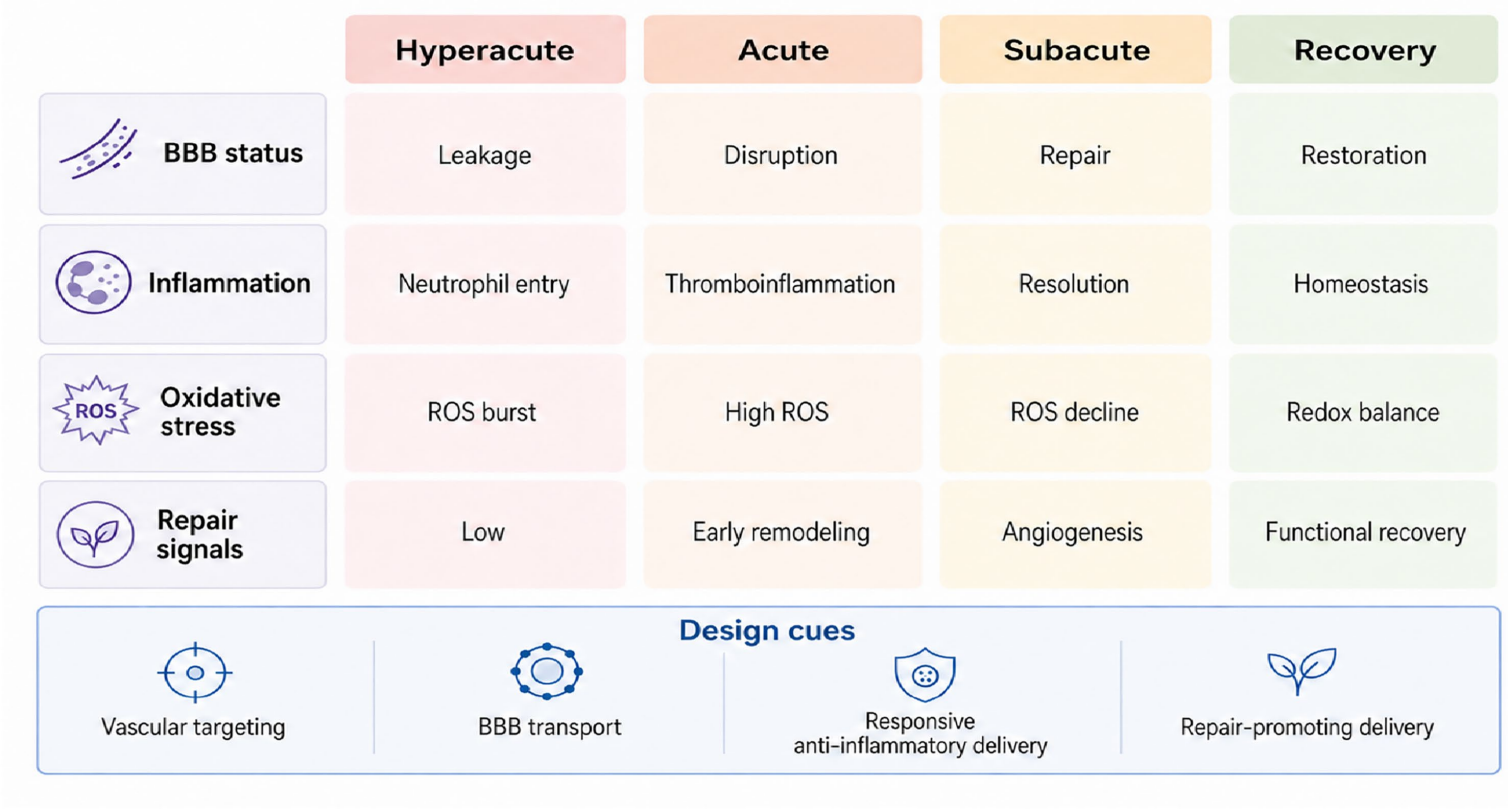
Spatiotemporal map of the ischemic microenvironment as a design brief. The hyperacute, acute, subacute, and recovery phases of IS are characterized by different BBB, thromboinflammatory, oxidative, immune, and repair cues. These cues define the design requirements for exosome-nanomaterial hybrids.

The ischemic microenvironment can therefore be translated into a staged design process. The clinical and pathological window should first be defined, followed by identification of the dominant cue within that window. An exosome source can then be selected to provide the desired biological interface, and a nanomaterial module can be added to supply the missing function, such as loading capacity, imaging contrast, magnetic guidance, or stimulus-responsive release. The final platform should be validated with assays that directly reflect the selected cue and the intended therapeutic function. The spatiotemporal organization of these pathological cues is summarized in Figure 1.

## 3 Engineering and functionalization of hybrid systems

**Figure 2:**
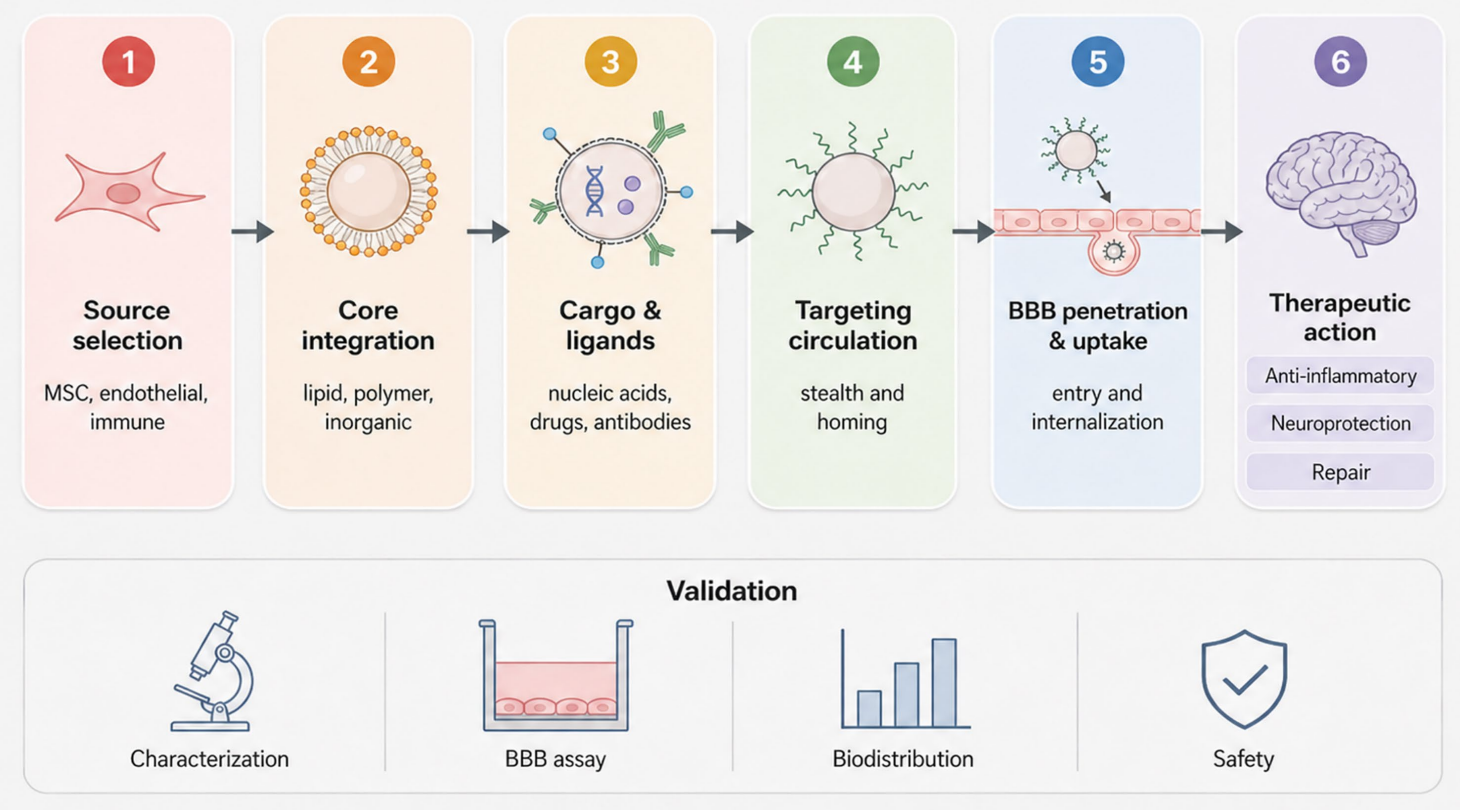
Lifecycle of a rationally designed exosome-nanomaterial hybrid nanoparticle. The design lifecycle includes modular assembly, systemic navigation, BBB interaction, ischemic lesion accumulation, microenvironment-responsive activation, cell-specific engagement, and mechanism-matched functional validation.

In this context, the engineering of exosome-nanomaterial hybrids should be guided by the dominant pathological features of the ischemic lesion rather than by material availability alone. A rational design process should first define the relevant pathological cue, then determine the appropriate exosome source, nanomaterial component, cargo loading strategy, surface modification, and functional validation method. This sequence is important because the suitability of a given material depends on the intended therapeutic purpose, such as BBB penetration, thromboinflammatory targeting, intracellular RNA delivery, imaging, or long-term neurovascular repair. A rational lifecycle for designing and validating exosome-nanomaterial hybrids is outlined in Figure 2.

### 3.1 Exosome sources and biological properties

Exosome source is not a trivial manufacturing variable but a primary determinant of biological behavior in hybrid systems. Parent-cell identity influences membrane composition, surface ligands, nucleic-acid cargo, uptake preference, and immunological signaling, thereby shaping whether a given exosomal shell is better suited for neuroprotection, vascular repair, inflammatory modulation, or BBB-oriented delivery (Liu et al., 2023; Hu et al., 2022; Yang and Chen, 2022; Wang et al., 2023). In ischemic stroke, the source classes most directly supported by current preclinical studies include mesenchymal stromal/stem cells (MSCs), neural stem or progenitor cells, endothelial cells, and selected immune-cell-derived vesicles.

Among these, mesenchymal-stem-cell-derived exosomes remain the most extensively studied source class in stroke models. Bone-marrow-mesenchymal-stem-cell exosomes reduced ischemic injury and improved neurological outcome in association with astrocytic IL-33/ST2 signaling (Liu et al., 2023). Other studies further showed that bone-marrow-derived exosomes promoted post-stroke angiogenesis through miR-21-5p-associated upregulation of VEGF/VEGFR2 and Ang-1/Tie-2 signaling (Hu et al., 2022), alleviated oxidative stress and inflammation through lncRNA ZFAS1-related mechanisms (Yang and Chen, 2022), and reduced mitochondrial injury and apoptosis through an exosomal KLF4/lncRNA-ZFAS1/FTO/Drp1 axis (Wang et al., 2023). In the reperfusion setting, mesenchymal-stem-cell extracellular vesicles also accumulated in injured brain regions after thrombolysis, attenuated BBB disruption and hemorrhagic transformation, and acted through a miR-125b-5p/TLR4/NF-κB pathway in astrocytes (Qiu et al., 2022). Human-induced-pluripotent-stem-cell-derived MSC extracellular vesicles likewise reduced infarct burden, improved behavioral recovery, and enhanced angiogenesis-related signaling in experimental stroke (Lu et al., 2023). More recently, human umbilical MSC-derived exosomes were shown to reprogram post-stroke microglial activation through an HMGB1–triggering receptor expressed on myeloid cells 1 (TREM1)–p38 mitogen-activated protein kinase (MAPK)-related mechanism, reinforcing the view that stromal source is biologically instructive rather than interchangeable (Zhang et al., 2025).

Neural-lineage exosomes form a second highly relevant source category when the desired biological profile emphasizes neuronal rescue, mitochondrial quality control, and endogenous repair. Neural-stem-cell-derived exosomes released from an adhesive hydrogel depot promoted cerebral angiogenesis and improved neurological recovery after ischemic stroke (Gu et al., 2023). Human neural-stem-cell-derived exosomes also protected against oxidative-stress-induced neuronal injury by activating the PTEN-induced kinase 1 (PINK1)/Parkin pathway, restoring mitophagy, improving mitochondrial function, and reducing apoptosis in ischemic models (Zhao et al., 2025). In parallel, human neural-progenitor-cell-derived exosomes carrying miR-100-5p reduced NOX4 expression, oxidative stress, endothelial apoptosis, and infarct size while protecting brain microvascular endothelial cells after acute ischemic injury (Zhao et al., 2025). These findings suggest that neural-lineage donors may be especially attractive when hybrid nanomedicine is intended to couple parenchymal neuroprotection with redox regulation and neurovascular remodeling (Gu et al., 2023; Zhao et al., 2025; Zhao et al., 2025).

Endothelial- and immune-cell-derived exosomes provide a more specialized but mechanistically important source class. Brain-microvascular-endothelial-cell-derived exosomes protected neurons from ischemia–reperfusion injury and promoted synaptic remodeling in mice (Sun et al., 2022). Follow-up work further showed that this source class improved transendothelial electrical resistance (TEER), reduced tracer permeability, restored tight-junction and basement-membrane proteins, and reinforced PDGF-PDGFRβ and Ang/Tie-2 signaling in MCAO/R models, underscoring its barrier-supportive properties (Sun J. et al., 2025). By contrast, immune-cell-derived exosomes are strongly state dependent: macrophage-derived exosomes produced under ischemia/reperfusion conditions exacerbated endothelial ferroptosis and barrier disruption through a THBS1-associated pathway (Liu et al., 2025). Taken together, these studies indicate that source selection for hybrid nanomedicine should be mechanism-matched rather than convenience-driven: stromal donors are currently best supported for scalable immunomodulation and vascular support, neural-lineage donors for neurorestorative and mitochondrial programs, endothelial donors for BBB-centric applications, whereas immune-derived vesicles require particularly strict control of donor activation state.

Taken together, current evidence supports the following practical framework for source selection. MSC-derived exosomes are currently the most generalizable choice when the design goal is scalable immunomodulation and tissue repair; neural-lineage exosomes are preferable when mitochondrial rescue, neurogenesis, or neuronal plasticity is central; endothelial exosomes are more rational for BBB-centric repair; and immune-cell-derived exosomes should be used only with strict control of donor activation state because they may either suppress or propagate inflammation. Thus, exosome source should be treated as a functional module rather than a passive membrane coating.

### 3.2 Nanomaterial components and hybridization strategies

Among currently reported brain-oriented hybrid systems, lipid-based components are the most frequently selected synthetic partners because they are membrane-compatible and support relatively gentle fusion, tunable loading, and straightforward surface engineering (Xie et al., 2025; Wang et al., 2024; Jiang et al., 2024; Evers et al., 2022; Lee et al., 2025; Son et al., 2025). In ischemic stroke, this design logic is directly illustrated by the recent Exo-Lip platform, in which neural-stem-cell-derived exosomes were fused with drug-loaded liposomes to generate a hybrid nanoparticle with improved stability and dual anti-inflammatory and metabolic effects in MCAO/R mice (Xie et al., 2025). Related central nervous system (CNS) studies show similar principles. Blood-exosome/tLyp-1-liposome hybrid nanovesicles enhanced BBB transcytosis and brain penetration in glioma models (Wang et al., 2024), whereas ROS-responsive exosome–liposome hybrids enabled dual nucleic-acid delivery with lesion-responsive activation in Alzheimer’s disease models (Jiang et al., 2024). These data support the interpretation that liposomal modules act not merely as passive depots but as structurally programmable membrane extenders that expand payload diversity while retaining part of the endogenous tropism of the exosomal component.

With respect to hybridization methodology, membrane fusion is the dominant engineering principle, but the implementation varies substantially in speed, loading mechanism, and structural preservation. Extrusion remains a widely used strategy. Extracellular-vesicle–liposome hybrids prepared by thin-film hydration and extrusion remained spherical, retained EV surface markers, encapsulated small interfering RNA (siRNA), and showed altered uptake and gene-silencing behavior across recipient cell types (Evers et al., 2022). In a glioblastoma-oriented brain-directed gene-delivery system, an electrostatically condensed plasmid DNA (pDNA)/polyamidoamine core was combined with exosome membranes through extrusion to generate a hybrid carrier with improved systemic brain delivery, and subsequent T7 peptide decoration further enhanced transfection (Lee et al., 2025). A more recent alternative is rapid fusogenic self-assembly. Cubosome–exosome membrane fusion completed by simple mixing within 10 min enabled near-complete loading of difficult macromolecules, including messenger RNA (mRNA), while preserving BBB uptake and transport behavior; importantly, the exosome-to-cubosome ratio itself influenced barrier penetration (Son et al., 2025). Together, these findings suggest that hybridization strategy should be matched to therapeutic format: extrusion is practical when structural consolidation and nucleic-acid condensation are priorities, whereas fusogenic lipid nanophases are especially attractive when rapid, non-destructive loading of large molecules is required (Evers et al., 2022; Lee et al., 2025; Son et al., 2025).

Beyond soft lipidic partners, inorganic and polymer-containing components broaden hybrid functionality toward imaging, magnetic guidance, photothermal intervention, and high-capacity gene packaging (Lee et al., 2025; Hu et al., 2024; Du et al., 2025). In post-stroke cognitive impairment, superparamagnetic iron oxide nanoparticles were incorporated into human mesenchymal-stem-cell-derived exosomes to generate magnetically targetable Spion-Ex, which improved brain accumulation, mitochondrial function, and behavioral recovery under magnetic guidance (Hu et al., 2024). Polymer-assisted formats offer a different advantage. In the pDNA/PHR-EM system, the exosome membrane was combined with a histidine/arginine-linked polyamidoamine core to stabilize plasmid complexation and enable systemic brain gene delivery (Lee et al., 2025). More recently, in an Alzheimer’s-disease-oriented multifunctional design, membranes from brain-microvascular-endothelial-cell exosomes and macrophage exosomes were hybridized around a polydopamine/resveratrol/aptamer-containing nanoparticle, thereby integrating BBB homing, inflammation tropism, molecular recognition, and photothermal responsiveness into a single construct (Du et al., 2025). Thus, the nanomaterial component in a hybrid system is chosen not only for loading capacity but also for the additional physical function it contributes, such as magnetic steering, imaging contrast, redox responsiveness, nucleic-acid condensation, or externally triggerable release.

Functionalization adds another layer of control beyond basic hybrid assembly. In pHybrid nanovesicles developed for glioma-oriented brain delivery, the tLyp-1-modified liposomal partner complemented blood-exosome-mediated BBB interaction and promoted deeper brain distribution (Wang et al., 2024). In ROS-responsive exosome–liposome nanovesicles designed for Alzheimer’s disease, angiopep-2 was incorporated to support lesion accumulation and therapeutic efficacy in the brain (Jiang et al., 2024). In the glioblastoma-directed pDNA/PHR-EM platform, T7 peptide installation increased gene delivery beyond that achieved by the unfunctionalized hybrid (Lee et al., 2025). Collectively, current engineering evidence suggests a practical design rule for stroke-oriented hybrid systems: lipidic fusion partners are favored when preserving BBB transport and membrane softness is critical; inorganic cores are useful when imaging or magnetic navigation is desired; polymeric condensers become valuable when plasmid or mRNA payloads are prioritized; and post-assembly ligand engineering should sharpen, rather than overwrite, exosome-derived tropism.

Taken together, current engineering evidence supports the following practical framework for material selection: Lipidic partners are most suitable when membrane compatibility, fusion, and flexible loading are prioritized; polymeric condensers are most useful for plasmid, siRNA, or mRNA packaging; inorganic components are justified when imaging contrast, magnetic steering, catalytic ROS modulation, or photothermal conversion is required; and hydrogel scaffolds are preferable for local retention or recovery-phase sustained release. A nanomaterial component should therefore be added only when it supplies a function that native exosomes cannot provide alone.

### 3.3 Cargo loading and surface engineering

Cargo loading determines not only what exosome-based or hybrid systems can deliver, but also how far these systems can move beyond the intrinsic bioactivity of native vesicles. In ischemic stroke and related brain-delivery studies, small molecules and protein therapeutics have often been incorporated through direct post-isolation approaches. Quercetin was loaded into blood-derived exosomes by ultrasonication and the vesicle surface was then conjugated with an anti-GAP43 antibody, yielding enhanced neuronal targeting and improved efficacy in MCAO/R rats (Guo et al., 2021). In a vascular-intervention-oriented example, exosome-coated tissue plasminogen activator (tPA)/catalase nanoformulations retained tPA activity, improved stability, achieved significant fibrinolysis, and added protection against hydrogen-peroxide-mediated oxidative stress (Khalil and Kanapathipillai, 2023). More recently, the Exo-Lip stroke platform fused neural-stem-cell-derived exosomes with liposomes preloaded with Yulangsan polysaccharide, illustrating how a synthetic partner can serve as a pre-engineered cargo reservoir while the exosomal component contributes biological compatibility and brain-directed function (Xie et al., 2025).

Nucleic-acid loading remains the most technically demanding but also the most transformative branch of exosome engineering. A foundational strategy is direct loading of exogenous RNA into isolated exosomes combined with genetically encoded targeting ligands. Alvarez-Erviti and colleagues established this paradigm by engineering donor cells to express Lamp2b fused to the rabies virus glycoprotein (RVG) peptide and then loading siRNA into purified exosomes, enabling systemic delivery of cargo to neurons, microglia, and oligodendrocytes in the mouse brain (Alvarez-Erviti et al., 2011). This logic was later extended to ischemic-stroke models. RVG-Lamp2b-modified exosomes loaded with miR-124 efficiently reached the infarct region and promoted cortical neurogenesis after ischemia (Yang et al., 2017), whereas RVG-targeted exosomes carrying HMGB1 siRNA reduced HMGB1 expression, inflammatory signaling, apoptosis, and infarct size more effectively than unmodified comparators (Kim et al., 2019). These studies collectively show that for brain diseases, cargo loading and targeting cannot be separated conceptually: nucleic-acid delivery becomes therapeutically meaningful only when the loaded vesicle is also engineered to engage the BBB and the diseased brain with sufficient selectivity (Alvarez-Erviti et al., 2011; Yang et al., 2017; Kim et al., 2019).

Hybrid systems further expand the loading design space because they decouple cargo capacity from the physical limitations of native exosomes alone. EV–liposome hybrid nanoparticles encapsulated siRNA, retained EV surface markers, and functionally delivered siRNA while preserving part of EV-derived biological activity (Evers et al., 2022). In an Alzheimer’s-disease-oriented study, ROS-responsive exosome–liposome hybrids were used to codeliver BACE1 siRNA and TREM2 plasmid, with BBB penetration and lesion accumulation enhanced by exosome homing and angiopep-2 assistance (Jiang et al., 2024). A different architecture, developed for brain gene delivery in glioblastoma, combined an electrostatically condensed plasmid DNA (pDNA)/polyamidoamine core with exosome membranes through extrusion, followed by T7 decoration, to improve systemic brain delivery of plasmid DNA (Lee et al., 2025). Hybridization itself can now serve as a loading technology. Extracellular vesicle-lipid nanoparticle (EV-LNP) fusion generated hybrid extracellular vesicles with successful mRNA loading, acquired endosomal-escape capacity, and functional *in vivo* mRNA delivery (Wang et al., 2025). Likewise, spontaneous hybridization of EVs with nucleic-acid-preloaded non-lamellar liquid-crystalline lipid nanoparticles enabled loading of multiple nucleic-acid types while maintaining accessibility and activity of EV membrane proteins (Bader et al., 2025). These findings are directly relevant to ischemic-stroke nanomedicine because they suggest that hybridization is not just a structural maneuver but an active solution to the long-standing loading bottleneck for exogenous RNAs and plasmids. Recent ischemic-stroke-oriented studies further support this concept, showing that erythrocyte membrane-fused plant-derived nanoparticles can serve as a gene therapy vehicle for cerebral ischemia/reperfusion injury, and that fused membrane-targeted nanoscale systems with asymmetric membrane structures can improve gene delivery for ischemic stroke (Li S. et al., 2025; Shi V. et al., 2025).

Surface engineering provides the second major lever of control. Broadly, two routes dominate: donor-cell engineering before vesicle collection and chemical or modular modification after vesicle isolation. Donor-cell engineering is exemplified by the RVG-Lamp2b platform, in which transfected donor cells generate vesicles bearing a targeting peptide that enables systemic delivery to neurons, microglia, and oligodendrocytes in the brain (Alvarez-Erviti et al., 2011). Post-isolation modification offers greater flexibility when the exosome source is fixed. Anti-GAP43-conjugated quercetin-loaded exosomes improved ischemic-neuron targeting after carbodiimide-based surface coupling (Guo et al., 2021). In hybrid platforms, post-assembly targeting can add a second targeting layer rather than replace exosomal identity: T7 decoration improved delivery in the glioblastoma-oriented pDNA/PHR-EM system (Lee et al., 2025), and angiopep-2 enhanced brain accumulation in ROS-responsive exosome–liposome nanovesicles designed for Alzheimer’s disease (Jiang et al., 2024). Overall, current evidence supports a practical design principle for stroke-oriented hybrid systems: cargo loading should be chosen according to payload class and intracellular-release requirements, whereas surface engineering should sharpen BBB transit, lesion localization, and cell-type selectivity without erasing the endogenous biological behavior that makes exosomes attractive in the first place.

**Figure 3:**
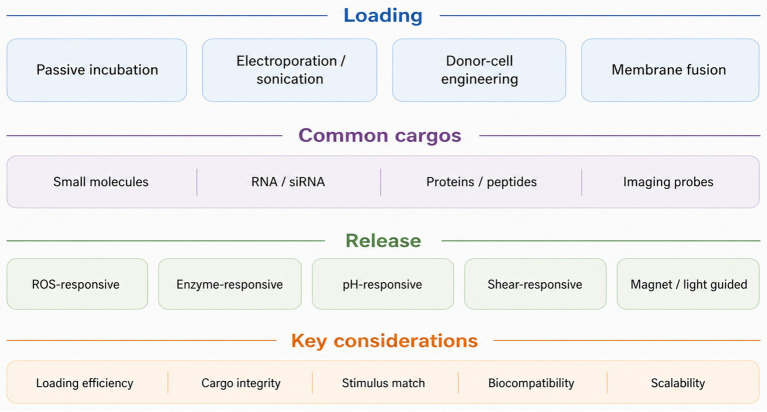
Cargo loading and controlled release strategies. Representative strategies include passive loading, active physical loading, donor-cell engineering, hybridization-based loading, and stimulus-responsive release. The optimal strategy should be selected according to cargo class and intracellular-release requirement.

Taken together, current design evidence supports the following practical framework for cargo selection: Acute IS platforms should prioritize antioxidant, anti-inflammatory, thromboinflammatory, or BBB-protective cargoes with rapid release; subacute platforms should prioritize microglial reprogramming, endothelial stabilization, or trophic signaling; recovery-phase systems should prioritize sustained delivery of pro-angiogenic, pro-neurogenic, or neuroplasticity-supportive cargoes. For nucleic-acid cargoes, intracellular release and endosomal escape should be evaluated directly rather than inferred from tissue accumulation alone. The major cargo-loading and controlled-release strategies are summarized in Figure 3.

### 3.4 Structural and functional characterization

Structural and functional characterization of hybrid systems should establish not only particle identity, but also successful hybridization, cargo incorporation, and preservation or gain of biological function. This is especially important because extracellular vesicles and synthetic nanoparticles differ substantially in size readout, counting accuracy, and phenotyping performance across analytical platforms, meaning that no single method can adequately capture hybrid-particle heterogeneity (Arab et al., 2021). In engineered EV systems, single-vesicle analyses further show that apparent bulk cargo enrichment can mask marked vesicle-to-vesicle heterogeneity, reinforcing the need for orthogonal characterization strategies when evaluating hybrid formulations (Silva et al., 2021). Accordingly, recent hybrid studies increasingly combine ensemble and single-particle methods rather than relying on size shift alone.

At the physicochemical level, the minimal evidence for a hybrid construct typically includes particle size distribution, polydispersity, zeta potential, morphology, retention of EV markers, and demonstration that the synthetic component is genuinely incorporated into the same particle population. EV–liposome hybrids have been characterized by EV marker profiling together with dynamic light scattering (DLS), nanoparticle tracking analysis (NTA), zeta-potential analysis, cryogenic transmission electron microscopy (cryo-TEM), and RNA encapsulation efficiency measurements (Evers et al., 2022). In mRNA-loaded hybrid extracellular vesicles generated by EV-LNP fusion, classical EV markers were retained, dual labeling and nanoflow cytometry identified hybridized particle subpopulations, and Raman analysis confirmed incorporation of LNP-derived material (Wang et al., 2025). In another nucleic-acid-loading study, spontaneous hybridization with non-lamellar lipid nanoparticles produced hybrid EVs in which a substantial subpopulation carried genetic cargo while EV membrane proteins remained surface-accessible and retained enzymatic activity (Bader et al., 2025). These studies indicate that hybrid characterization has moved beyond simple size confirmation toward multiparametric verification of co-assembly and retained biointerface integrity (Evers et al., 2022; Wang et al., 2025; Bader et al., 2025).

Demonstrating membrane fusion itself is another critical layer of characterization, especially for exosome–lipid hybrids. In the cubosome–exosome platform, fusion was validated through fluorescence resonance energy transfer (FRET) kinetics, time-resolved small-angle X-ray scattering (SAXS), DLS, fluorescence colocalization, and cryo-TEM, providing direct evidence that membrane mixing and structural reorganization had occurred rather than simple particle co-incubation (Son et al., 2025). Notably, the exosome-to-cubosome ratio affected both fusion behavior and downstream BBB uptake and transport, highlighting that characterization parameters can themselves become functional design variables (Son et al., 2025). These observations are consistent with broader single-particle evidence that different EV subpopulations do not behave identically during analysis or engineering (Arab et al., 2021; Silva et al., 2021).

Functional characterization should then determine whether hybridization improves delivery performance without erasing the beneficial biological attributes of the original exosome source. In EV–liposome hybrids, formulation-dependent uptake, toxicity, and gene-silencing efficacy varied across recipient cell types, indicating that hybrid composition directly alters downstream biological performance (Evers et al., 2022). In the mRNA-focused hybrid EV study, the key gain of function was not merely higher loading but acquisition of endosomal-escape capability and functional *in vivo* mRNA delivery, which source EVs alone did not provide (Wang et al., 2025). In fusogenic cubosome–exosome hybrids, large molecules including mRNA were loaded with very high efficiency while BBB uptake and transport behavior were preserved and the loaded cubosome intermediates remained structurally stable for weeks at room temperature (Son et al., 2025). These data suggest that functional characterization should include intracellular release behavior, preservation of source-vesicle bioactivity, and formulation stability in addition to conventional uptake assays (Evers et al., 2022; Son et al., 2025; Wang et al., 2025).

For brain-directed hybrids, characterization is incomplete unless it is linked to BBB-relevant and disease-relevant performance. In pHybrid nanovesicles, assessment extended to BBB transcytosis into brain parenchyma and subsequent target-cell uptake, showing that hybridization changed not only composition but also trafficking behavior in the brain-delivery context (Wang et al., 2024). In the cubosome–exosome study, a three-dimensional BBB model containing endothelial cells, astrocytes, and pericytes was used to quantify hybrid uptake and transport after barrier maturation had been verified by junctional markers (Son et al., 2025). In magnetically targetable iron-oxide exosomes, TEM and NTA were complemented by MRI and near-infrared fluorescence imaging to demonstrate increased brain delivery under magnetic guidance (Hu et al., 2024). Finally, in the recent Exo-Lip ischemic-stroke study, hybrid characterization was further linked to infarct reduction, behavioral improvement, and transcriptomic evidence of pathway modulation *in vivo* (Xie et al., 2025). Thus, for exosome–nanomaterial hybrids aimed at ischemic stroke, the most informative characterization workflow is one that moves from particle identity and fusion verification to cargo function, BBB transport, lesion accumulation, and biological efficacy in a connected manner (Xie et al., 2025; Wang et al., 2024; Son et al., 2025; Hu et al., 2024; Wang et al., 2025; Bader et al., 2025; Arab et al., 2021; Silva et al., 2021).

Characterization of a stroke-oriented hybrid formulation should integrate particle identity, hybridization evidence, cargo behavior, and disease-relevant function. Particle identity and purity should be established first, followed by evidence that the exosomal and synthetic components form the intended functional construct. Cargo loading, release behavior, BBB interaction, lesion targeting, and therapeutic activity should then be assessed using assays that reflect the intended mechanism. MISEV2023 provides a useful baseline for EV characterization, but hybrid systems require additional evidence that the biological and synthetic components behave as one functional delivery platform (Welsh et al., 2024).

## 4 Therapeutic and theranostic applications in ischemic stroke

The therapeutic value of exosome-based hybrid nanomedicine in ischemic stroke lies in its ability to intervene across multiple stages of injury evolution rather than targeting a single pathological event. Following cerebral ischemia–reperfusion, the brain undergoes a dynamic progression involving acute oxidative stress, excitotoxicity, blood–brain barrier disruption, thromboinflammatory amplification, secondary immune-cell recruitment, and, subsequently, tissue remodeling and neurovascular repair. Accordingly, different exosome/EV-based hybrid systems have been developed to address distinct yet overlapping therapeutic goals, including acute neuroprotection, modulation of microglia/macrophage and neutrophil-driven inflammation, imaging-guided delivery and stimulus-responsive release, and promotion of angiogenesis, neurogenesis, and functional recovery. Importantly, the biological effects of these platforms are shaped not only by their parent-cell or parent-organism origin, but also by hybrid engineering strategies such as hydrogel integration, membrane fusion, magnetic or metallic functionalization, surface ligand decoration, and cargo enrichment (Table 1).

**Table 1:** Representative exosome-/EV-based hybrid and related nanosystems for ischemic stroke and brain delivery.

Tier	Model/evidence	EV/exosome source	Nanocomponent/material	Cargo	Engineering strategy	Main function/key findings	Ref
Strict/material-assisted hybrid	tMCAO rat; *in vivo*	MSC-derived extracellular nanovesicles (NVs)	Iron oxide nanoparticles (IONPs) + external magnetic field	Intrinsic EV cargo enriched after IONP priming	IONP-harboring MSCs used to produce magnetic NVs; systemic injection + magnetic navigation	5.1-fold higher lesion localization; enhanced anti-inflammation, angiogenesis, anti-apoptosis; reduced infarct size and improved motor recovery	Kim et al. (2020)
Strict/material-assisted hybrid	Type 2 diabetic stroke mouse; in vivo + *in vitro*	Neural stem cell-derived EVs	Glucose/ROS dual-responsive hydrogel (PBA-modified HA + PVA)	Intrinsic EV cargo	EVs incorporated into injectable self-healing hydrogel for microenvironment-responsive sustained release	Improved EV retention, angiogenesis and neurobehavioral recovery in diabetic stroke	Jiang et al. (2022)
Strict/material-assisted hybrid	MCAO mouse; in vivo	Neural stem cell-derived exosomes	Adhesive HAD hydrogel	Intrinsic exosomal cargo	Exosome-loaded adhesive hydrogel with controlled release after stereotactic delivery	Promoted angiogenesis, reduced inflammation and improved neurological function	Gu et al. (2023)
Strict/material-assisted hybrid	In vitro thrombolysis/H2O2-protection assays; not a direct stroke-efficacy model	Human brain microvascular endothelial cell exosomes	Surface-conjugated tPA ± catalase nanoformulation	tPA; catalase	EDC/NHS-mediated exosome surface coating/conjugation	Retained tPA activity, improved fibrinolysis and reduced H2O2-mediated toxicity; useful as thrombolytic nanoplatform	Khalil and Kanapathipillai (2023)
Strict/material-assisted hybrid	Ischemic stroke model; in vivo + intranasal delivery	Grapefruit-derived EVs	Borneol-modified biomimetic nanoparticles carrying Xingnaojing microemulsion	Xingnaojing microemulsion	Bo-GEV membrane-assisted assembly of intranasal biomimetic nanoparticles	Improved brain targeting; anti-apoptotic, anti-inflammatory, antioxidant, mitochondrial and BBB-protective effects	Li et al. (2024)
Strict/material-assisted hybrid	Ischemic stroke rat; imaging/biodistribution	Human MSC-derived EVs	Ultrasmall superparamagnetic iron oxide (USPIO/SPION)	No added therapeutic cargo	Sonication-based iron oxide labeling for MRI tracking	Enabled MRI visualization of EV biodistribution/localization in ischemic stroke	Helsper et al. (2024)
Strict/material-assisted hybrid	Ischemic stroke rat; in vivo therapeutic assessment + MRI	Hypoxia-preconditioned human MSC-derived EVs	Iron oxide labeling for MRI	Hypoxia-enriched EV proteins/miRNAs	Hypoxia preconditioning to raise EV yield + iron oxide labeling for imaging readout	57% higher EV yield; lesion localization on MRI; therapeutic response after a single bolus injection	Helsper et al. (2025)
Strict/material-assisted hybrid	Stroke mouse; in vivo + multimodal imaging	M2 microglia-derived EVs	Polydopamine surface layer + RVG29 + multimodal imaging probes	Intrinsic M2-EV cargo (no added therapeutic drug)	Dopamine surface functionalization enables one-step peptide/probe conjugation	Improved neuronal targeting, whole-body-to-cellular imaging, and reduced neuronal apoptosis	Shi X. et al. (2025)
Strict/material-assisted hybrid	MCAO/R model; in vitro + in vivo gene delivery	Bone marrow MSC exosomes	AMD3100-modified targeted lipid calcium phosphate (T-LCP)	miRNA-210	Membrane fusion of EXO with T-LCP to form EXO-T-LCP	Combined exosome biocompatibility with CXCR4-targeted gene delivery to ischemic lesions	Shi et al. (2025)
Strict/material-assisted hybrid	MCAO/R; in vitro + in vivo	Neural stem cell-derived exosomes	Liposomes fused with exosomes (Exo-Lip)	Yulangsan polysaccharide	Hybrid exosome-liposome fusion plus drug loading	Dual modulation of neuroinflammation and lipid metabolism; improved stability and therapeutic efficacy	Xie et al. (2025)
Strict/material-assisted hybrid	CI/R/tMCAO mouse; in vitro + in vivo	Panax notoginseng-derived exosome-like nanoparticles (PDNs)	Erythrocyte membrane fusion	Agomir-124	RBC membrane fusion to form R-PDN, then gene loading (Ago124@R-PDN)	Extended half-life from 7 min to 11.3 h; improved brain delivery, infarct reduction and functional recovery	Li et al. (2025)
Strict/material-assisted hybrid	Photothrombotic stroke mouse + endothelial/zebrafish assays	Ligusticum sinense chuanxiong-derived EVs (CXEVs)	Plant EV nanoplatform; engineered G3702@CXEVs	Intrinsic plant EV cargo ± optional G3702 co-loading	Native brain-penetrant plant EVs with secondary hydrophobic cargo loading	Metabolic reprogramming + VEGF-linked vascular repair; G3702@CXEVs further enhanced neurogenesis and angiogenesis	Wang et al. (2026)
Broad related EV-based nanosystem	MCAO rat + oxidative-stress NSC model	Human neural stem cell-derived exosomes	No separate nanomaterial	BDNF	BDNF loading into NSC exosomes	Enhanced microglial suppression, NSC neuronal differentiation and post-stroke recovery vs. native exosomes	Zhu et al. (2023)
Broad related EV-based nanosystem	Ischemic stroke mouse; intranasal	MSC-derived small EVs	No separate nanomaterial	BDNF	Parental MSC engineering/BDNF-enriched sEVs for intranasal nose-to-brain delivery	Improved functional behavior, neurogenesis, angiogenesis, synaptic plasticity and anti-inflammatory effects	Zhou et al. (2023)
Broad related EV-based nanosystem	tMCAO/CI-R rat; in vivo + in vitro	Panax notoginseng-derived exosome-like nanoparticles (PDNs)	Plant-derived ELN platform (no extra material)	Intrinsic lipids/plant cargo	Isolation of native plant ELNs with no added material modification	Crossed BBB without modification; reduced infarct volume; shifted microglia toward M2 phenotype via PI3K/Akt	Li et al. (2023)
Broad related EV-based nanosystem	Ischemic stroke/reperfusion injury; in vitro + in vivo	MSC-derived apoptotic vesicles (ApoVs)	MAP targeting peptide on ApoV surface	α-Mangostin	Drug loading during apoptosis induction + microenvironment-responsive targeting modification	Targeted ischemic brain delivery, inflammatory regulation and tissue repair	You et al. (2023)
Broad related EV-based nanosystem	Post-stroke cognitive impairment model	HT22 hippocampal cell-derived EVs	No separate nanomaterial	Adenosine	Adenosine encapsulation into hippocampal-cell EVs (Ev-Ad)	Targeted damaged hippocampus, reduced hippocampal cell death/inflammation, improved learning and memory after stroke	Zhang et al. (2024)
Broad related EV-based nanosystem	MCAO mouse + OGD/R microglia	Adipose-derived stem cell exosomes	No separate nanomaterial; peptide-engineered exosome membrane	Intrinsic anti-ferroptosis cargo	Lamp2b/M2pep engineering to generate M2pep-ADSC-Exo targeting M2 microglia	Reduced M2 microglia ferroptosis, improved inflammatory milieu and neurological recovery	Wang et al. (2024)
Broad related EV-based nanosystem	tMCAO mouse; in vitro + in vivo	Neural stem cell-derived EVs	No separate nanomaterial; bio-click surface functionalization	VEGF	RGD peptide display + VEGF loading (dual-functionalized EVs)	5.2-fold higher endothelial uptake; reduced infarct volume; promoted angiogenesis and neurogenesis	Shi et al. (2025)
Broad related EV-based nanosystem	Ischemic stroke; in vivo	*Lactobacillus reuteri*-derived extracellular vesicles	Bacterial EV platform (no added material)	Intrinsic bacterial EV cargo	Use of naturally brain-targeting probiotic EVs	Targeted TLR2-upregulated injured brain; scavenged ROS; suppressed immune infiltration and BBB damage	Sun et al. (2025)
Broad related EV-based nanosystem	MCAO rat + OGD/R HT22	*Houttuynia cordata*-derived extracellular vesicle-like particles	Plant EV-like particles (no added material)	Endogenous miR159a	Native plant EVLP isolation	Crossed into infarct area, preserved BBB, reduced infarct volume and neuronal ferroptosis via ACSL4 targeting	Zhang et al. (2025)
Broad related EV-based nanosystem	tMCAO rat + OGD/R HT22	*Momordica charantia* small EVs	Plant sEV platform (no added material)	Endogenous miR-5813b	Native MC-sEV isolation	Mitigated neuronal ferroptosis by suppressing TRIM62-mediated GPX4 ubiquitination; good biosafety	Huang et al. (2025)
Broad related EV-based nanosystem	Stroke models in vitro + in vivo	Panax notoginseng-derived extracellular-like nanoparticle vesicles (NotoEV)	Plant EV platform (no added material)	Cross-kingdom plant miRNAs	Optimized high-yield extraction and screening across medicinal-plant PEVs; NotoEV selected as best performer	Suppressed stress-granule nucleators, activated mTOR/Bcl-2-TOM20 pathways, reduced infarct volume and restored neuronal function	Yu et al. (2026)

Analysis of the platforms summarized in Table 1 reveals several design trends. Lipid-based hybrids and hydrogel-integrated systems dominate when the goal is anti-inflammatory therapy or sustained repair, whereas inorganic modules are used mainly for imaging, magnetic guidance, or catalytic activity. However, relatively few systems integrate more than two microenvironment-responsive features in one platform, and many sophisticated theranostic architectures remain better established in adjacent CNS disease models than in stroke itself. This gap highlights the need for a more explicit design matrix linking therapeutic objective, pathological cue, exosome source, nanomaterial module, and validation endpoint. To translate these observations into a practical design guide, Table 2 further summarizes therapeutic objectives, dominant pathological cues, recommended EV sources, nanomaterial modules, functionalization strategies, validation endpoints, and translational concerns.

**Table 2:** Design principle and selection matrix for exosome-nanomaterial hybrids in ischemic stroke.

Therapeutic objective	Dominant pathological cue	Recommended EV/exosome source	Recommended nanomaterial module	Functionalization/cargo logic	Key validation endpoints	Main translational concern
BBB penetration and lesion targeting	BBB transport remodeling, endothelial activation, regional permeability	Endothelial, neural-lineage or MSC-derived exosomes	Lipidic hybrid, magnetic particle, or imaging-compatible module	BBB transport ligand, ischemic-vessel targeting ligand, or physical guidance	TEER, tracer permeability, tight-junction proteins, brain biodistribution, lesion-to-organ ratio	Avoid excessive BBB opening and off-target organ accumulation
Acute neuroprotection	ROS, excitotoxicity, mitochondrial dysfunction	Neural-lineage or MSC-derived exosomes	ROS-scavenging nanozyme, antioxidant polymer, or liposome reservoir	Antioxidant, anti-apoptotic, mitochondrial-protective cargo	Infarct volume, neuronal survival, ROS, mitochondrial markers, early neurological scores	Therapeutic window and rapid release kinetics
Thromboinflammatory control	Platelet activation, neutrophil recruitment, NETosis, endothelial adhesion	Platelet-derived, MSC-derived, or engineered exosomes with vascular tropism	Shear-, ROS-, elastase-, or protease-responsive module	Anti-NETosis, anti-inflammatory, or antiplatelet-compatible cargo	MPO-DNA, Cit-H3, platelet–neutrophil aggregates, cytokines, recanalization-compatible safety	Interaction with thrombolysis, anticoagulation, or platelet function
Microglial reprogramming	M1-like activation, ferroptosis, inflammasome signaling	MSC, adipose-derived MSC, or microglia-targeted engineered exosomes	Lipid/polymer hybrid suitable for RNA or small-molecule delivery	Mannose, M2pep, CD86-targeting peptide, anti-ferroptotic or anti-inflammatory cargo	Microglial phenotype, cytokines, ferroptosis markers, neuronal survival	Cell-state specificity and immune over-suppression
Imaging-guided therapy	Uncertain lesion delivery, heterogeneous retention, need for dose guidance	MSC or neural-lineage exosomes compatible with labeling	Iron oxide, Prussian blue, NIR, photoacoustic, or magnetic module	Contrast agent plus therapeutic cargo; externally triggered release if justified	MRI/NIR/photoacoustic signal, co-localization with lesion, release confirmation, efficacy	Added material toxicity and regulatory complexity
Recovery and neurovascular repair	Angiogenesis, neurogenesis, synaptic remodeling, BBB reconstruction	Neural stem cell, MSC, endothelial or preconditioned exosomes	Hydrogel depot, adhesive scaffold, or sustained-release hybrid	BDNF, pro-angiogenic miRNA, trophic cargo, matrix-retentive design	Microvessel density, neurogenesis, synaptic markers, BBB repair, long-term behavior	Invasiveness, retention duration, and chronic safety
Product translation	Batch variability, active subpopulation uncertainty, manufacturing scale-up	Source selected for reproducibility and potency	Material selected for scalable assembly and validated removal of free component	Release-testable potency assay linked to mechanism	Identity, purity, potency, stability, sterility, biodistribution, toxicology	Classification as biologic, nanomedicine, combination product, or engineered EV therapy

This matrix is intended as a design guide rather than a rigid classification. EVs, extracellular vesicles; IS, ischemic stroke; BBB, blood–brain barrier; ROS, reactive oxygen species; NETosis, neutrophil extracellular trap formation; TEER, transendothelial electrical resistance.

Each application discussed below is therefore evaluated through four questions: which IS stage is being targeted, which microenvironmental cue is being exploited, which exosomal and synthetic modules execute the intended function, and which assays prove that the design works through the proposed mechanism.

### 4.1 BBB-penetrant delivery and ischemia-targeted accumulation

In ischemic stroke, effective exosome-mediated delivery is increasingly understood not as a passive consequence of barrier leakage, but as an actively engineered process of BBB traversal, lesion localization, and target-cell engagement. Foundational evidence for this concept came from RVG-decorated exosome systems. Yang et al. showed that exosomes expressing RVG-Lamp2b efficiently delivered miR-124 to the infarct site after systemic administration, thereby promoting cortical neurogenesis and protecting against ischemic injury (Yang et al., 2017). In a related stroke study, Kim et al. demonstrated that RVG-modified exosomes carrying HMGB1 siRNA reduced infarct size more effectively than unmodified controls, further validating receptor-assisted brain delivery as a practical strategy for targeting the ischemic brain after intravenous administration (Kim et al., 2019). Together, these studies established that engineered exosomes can be directed across the BBB and into the ischemic territory with therapeutically meaningful selectivity, rather than relying solely on nonspecific post-stroke permeability.

Subsequent work expanded this principle from brain entry toward more context-aware localization within the neurovascular unit. Guo et al. loaded quercetin into exosomes and surface-conjugated an anti-GAP43 antibody, generating a dual-targeting formulation that preferentially delivered cargo toward impaired neurons and significantly improved functional recovery after MCAO/R (Guo et al., 2021). Qiu et al. further showed that mesenchymal stem cell-derived extracellular vesicles attenuated tPA-induced BBB disruption and hemorrhagic transformation in murine ischemic stroke models, positioning EVs as reperfusion adjuvants in vascularly vulnerable settings (Qiu et al., 2022). In a more explicitly cascade-targeted design, Liu et al. engineered mannose-conjugated luteolin-loaded platelet-derived exosomes, showing that because platelets naturally gather in pathological ischemic cerebral vessels, the exosomal platform first localized to ischemic vasculature and subsequently enhanced microglial uptake through mannose-receptor interactions (Liu et al., 2024). These studies indicate that in stroke, lesion accumulation can be improved by exploiting disease-specific vascular adhesion, damaged-neuron recognition, or reperfusion-associated BBB vulnerability rather than by treating the ischemic brain as a uniformly accessible compartment.

An additional advance has been the shift from regional targeting alone to cell-selective accumulation within the ischemic niche. Wang et al. developed M2pep-modified adipose-derived stem cell exosomes (M2pep-ADSC-Exo) and showed that the engineered vesicles exhibited higher uptake by M2 microglia than non-targeted ADSC-Exo *in vitro*, and after intranasal administration to MCAO mice, produced higher PKH-26/CD206 double-positive signals *in vivo*, indicating more efficient accumulation in M2 microglia in the ischemic penumbra (Wang et al., 2024). A complementary but mechanistically distinct strategy was reported by Yang et al., who engineered exosomes with a peptide that selectively binds CD86-positive microglia and loaded them with quercetin; their Que.@micro-Exo formulation accumulated in CD86-positive microglia in ischemic regions, reduced off-target reticuloendothelial accumulation, and improved post-stroke functional recovery (Yang et al., 2025). More recently, Zhang et al. showed that intranasally administered human umbilical MSC-derived exosomes (hUMSC-Exos) accumulated in the mouse brain after tMCAO and reprogrammed microglial inflammatory signaling through the HMGB1–TREM1–p38 MAPK axis, supporting the idea that brain accumulation and cell-state-specific reprogramming can be functionally coupled in the same platform (Zhang Z. et al., 2025). Collectively, these results suggest that the most effective ischemia-targeted systems are likely those that combine regional lesion entry with secondary cell-type selectivity inside the post-stroke microenvironment.

Hybrid and exosome-integrated nanoplatforms have further extended the toolbox for BBB-penetrant delivery. Kim et al. reported one of the clearest stroke-specific demonstrations of navigation-enhanced targeting by generating mesenchymal-stem-cell-derived magnetic extracellular nanovesicles; after systemic administration in tMCAO rats, magnetic guidance increased localization to the ischemic lesion by 5.1-fold and substantially improved therapeutic outcome (Kim et al., 2020). In a more recent stroke-tailored hybrid design, Xie et al. developed exosome-liposome hybrid nanoparticles (Exo-Lip) for ischemic stroke and explicitly leveraged the BBB permeability and intrinsic anti-inflammatory activity of the exosomal component while adding synthetic-liposome-based antioxidant and immunoregulatory capacity (Xie et al., 2025). Although direct biodistribution studies of stroke-specific exosome–nanomaterial hybrids are still fewer than those of engineered exosomes alone, the available evidence already supports a clear design principle: in ischemic stroke, BBB penetration and ischemia-targeted accumulation are most convincing when the biological navigation of exosomes is combined with source-matched targeting, lesion-responsive retention, or auxiliary physical guidance rather than being attributed to passive leakage alone.

For BBB-penetrant and ischemia-targeted delivery, the most defensible platforms are those that combine a brain- or lesion-facing biological interface with an independently measurable targeting mechanism. Relevant strategies include receptor-assisted BBB transport, activated-vascular adhesion, magnetic navigation, intranasal nose-to-brain transport, and focused-ultrasound-assisted delivery. Intranasal brain-derived neurotrophic factor (BDNF)-loaded small EVs support the feasibility of a non-invasive route for cerebral ischemia therapy, although this approach still requires careful assessment of dosing reproducibility and regional brain distribution (Zhou et al., 2023).

### 4.2 Neuroprotection and thromboinflammatory modulation

The therapeutic relevance of exosome-based nanomedicine in ischemic stroke lies not only in brain delivery, but also in its capacity to couple neuroprotection with active remodeling of the post-ischemic inflammatory milieu. In relatively early but still informative studies, bone-marrow-mesenchymal-stem-cell-derived exosomes reduced infarct volume and improved neurological outcomes in MCAO mice while enhancing IL-33 and ST2 expression in the peri-infarct region, and *in vitro* experiments further indicated that their neuroprotective effect depended on astrocytic IL-33/ST2 signaling (Liu et al., 2023). Complementing this anti-injury mechanism, bone-marrow-derived exosomes were also shown to promote angiogenesis after cerebral ischemia through miR-21-5p-associated upregulation of VEGF/VEGFR2 and Ang-1/Tie-2 signaling (Hu et al., 2022), while human induced pluripotent stem cell-derived MSC (hiPSC-MSC)-derived extracellular vesicles reduced infarct burden, improved spontaneous motor behavior, and enhanced VEGF/CXCR4-associated angiogenesis in experimental stroke models (Lu et al., 2023). Together, these studies suggest that exosome-mediated neuroprotection in stroke is not restricted to direct neuronal rescue, but also involves restoration of trophic and vascular support programs that can reshape the injured neurovascular niche (Liu et al., 2023; Hu et al., 2022; Lu et al., 2023).

More recent work has shifted from generic neuroprotection toward explicit modulation of the inflammatory microenvironment, especially microglial polarization. Liu et al. designed mannose-conjugated, luteolin-loaded platelet-derived exosomes as a cascade-targeting platform that first localized to ischemic vessels and then targeted microglia; this formulation inhibited detrimental M1 polarization, promoted an anti-inflammatory M2 shift, reduced infarct size, rescued BBB damage, and prevented inflammatory transformation of astrocytes (Liu et al., 2024). Wang et al. further showed that adipose-derived-stem-cell exosomes decreased the susceptibility of M2 microglia to ferroptosis through an Fxr2/Atf3/Slc7a11-related mechanism, thereby suppressing the inflammatory microenvironment and promoting neuronal survival; after additional engineering with M2pep, the targeted exosomes more effectively inhibited M2 microglial ferroptosis and improved neurological recovery in MCAO mice (Wang et al., 2024). These findings are notable because they connect exosome therapy to a more nuanced view of thromboinflammation, in which preservation of anti-inflammatory microglial states may secondarily restrain the propagation of vascular and parenchymal injury (Wang et al., 2024; Liu et al., 2024).

The field has also moved toward targeting discrete pathogenic inflammatory cell states rather than broadly suppressing inflammation. Yang et al. identified p21 + CD86 + microglia as a previously underappreciated pro-inflammatory microglial population accumulating in ischemic regions, and engineered a quercetin-loaded exosome formulation that selectively targeted these cells (Yang et al., 2025). Systemic administration of this construct reduced p21 + CD86 + microglia, suppressed their pro-inflammatory phenotype, mitigated BBB disruption, promoted beneficial microglial polarization, decreased neutrophil infiltration, and improved functional recovery after cerebral ischemia (Yang et al., 2025). In parallel, Zhang et al. demonstrated that intranasally delivered hUMSC-Exos selectively accumulated in ischemic regions, reduced neuronal apoptosis, and promoted a sustained shift of microglia toward an anti-inflammatory phenotype; mechanistically, they delineated an HMGB1–TREM1–p38 MAPK axis in which hUMSC-Exos downregulated TREM1-dependent NF-κB/p38 MAPK signaling, thereby suppressing microglial activation, migration, and proliferation (Zhang Z. et al., 2025). These studies provide some of the clearest evidence to date that exosome engineering can be used not merely to blunt inflammation in general, but to reprogram specific inflammatory states that drive secondary neurovascular damage in stroke (Zhang Z. et al., 2025; Yang et al., 2025).

Hybrid systems now extend this logic by integrating exosome-derived biological activity with synthetic nanocomponents that amplify anti-inflammatory and antioxidant performance. In the recent Exo-Lip study, neural-stem-cell-derived exosomes were fused with Yulangsan-polysaccharide-loaded liposomes, generating a hybrid platform that decreased tumor necrosis factor *α* (TNF-α) and IL-6, increased IL-10 and transforming growth factor *β* (TGF-β), alleviated oxidative stress, restored lipid metabolism, reduced infarct volume, and improved both motor and cognitive outcomes in MCAO/R mice (Xie et al., 2025). Transcriptomic analysis further linked these effects to inflammatory and lipid-regulatory networks, including AKT/Nrf2/HO-1 signaling (Xie et al., 2025). At the same time, donor source and pathological state remain critical determinants of whether exosomes suppress or exacerbate post-stroke inflammation: exosomes released from hypoxic vascular smooth muscle cells promoted M1 microglial polarization through Src transfer, whereas Src inhibition reversed this phenotype and improved neuronal outcome in permanent middle cerebral artery occlusion (pMCAO) mice (Zhang X. et al., 2024). Pathogenic exosomal signaling can also extend to the vascular compartment: macrophage-derived exosomes enriched in THBS1 aggravated cerebral ischemia–reperfusion injury by inducing endothelial ferroptosis and barrier disruption (Liu et al., 2025). Plant-derived and bacterial extracellular-vesicle-based platforms have also expanded the therapeutic design space for ischemic stroke. For example, Panax notoginseng-derived exosome-like nanoparticles attenuated ischemia–reperfusion injury by altering microglial polarization, Houttuynia cordata Thunb-derived extracellular-vesicle-like particles alleviated ischemic brain injury through miR159a-mediated ACSL4 targeting and ferroptosis suppression, and brain-targeting bacterial extracellular vesicles enhanced ischemic stroke therapy through efficient ROS elimination and suppression of immune infiltration (Li S. et al., 2023; Zhang S.Y. et al., 2025; Sun M. et al., 2025). Taken together, current evidence supports a practical conclusion for stroke nanomedicine: the most convincing neuroprotective exosome-based systems are those that simultaneously reduce neuronal loss and reshape thromboinflammatory circuits—primarily through microglial reprogramming, BBB-associated inflammatory control, and attenuation of secondary immune-cell recruitment—while direct exosome-mediated targeting of classical NETosis-driven immunothrombosis remains an emerging rather than fully established strategy.

For neuroprotection and thromboinflammatory modulation, the selected platform should match the inflammatory cell state being targeted. When the main objective is to regulate microglial polarization, microglia-recognition ligands, anti-ferroptotic cargoes, or anti-inflammatory nucleic acids may be prioritized. When the target is NETosis or vascular immunothrombosis, platelet- or neutrophil-facing membranes, ROS- or elastase-responsive linkers, and anti-NETosis cargoes may be more appropriate. These choices should be supported by mechanism-specific assays rather than by broad behavioral improvement alone.

### 4.3 Imaging-guided therapy and stimulus-responsive systems

Imaging-guided and stimulus-responsive exosome systems remain less mature in ischemic stroke than in exosome-mediated neuroprotection or inflammatory modulation, yet the field is now moving from passive biodistribution tracking toward actively guided delivery and lesion-responsive activation. In stroke itself, one of the clearest recent demonstrations comes from low-intensity pulsed focused ultrasound-assisted exosome delivery. Alptekin et al. showed that focused ultrasound increased the accumulation of endothelial-progenitor-cell-derived exosomes in the ischemic region without increasing albumin leakage or cluster of differentiation 45-positive (CD45+) cell accumulation, while the combination of ultrasound and endothelial progenitor cell (EPC)-derived exosomes reduced stroke volume and increased neovascularization around the lesion (Alptekin et al., 2025). Imaging-enabled stroke studies are also becoming more rigorous at the formulation level. Helsper et al. reported that ultrasmall superparamagnetic iron oxide nanoparticle-labeled human MSC-derived EVs (hMSC-EVs) could be visualized by MRI in an ischemic-stroke model (Helsper et al., 2024), and a subsequent study from the same group further showed that iron-oxide-labeled hypoxia hMSC-EVs localized to the ischemic lesion and evoked a therapeutic response that could be assessed by MRI and metabolic readouts (Helsper et al., 2025). In a related stroke-associated context, Hu et al. developed superparamagnetic-iron-oxide-loaded human MSC-derived exosomes for post-stroke cognitive impairment and showed that magnetic attraction enhanced BBB penetration, brain targeting, neuronal mitochondrial rescue, and behavioral improvement (Hu et al., 2024). Together, these studies suggest that in stroke, imaging-guided exosome therapy is currently strongest where imageable inorganic labeling and external physical steering are directly integrated into the delivery workflow.

Adjacent CNS disease models provide a broader view of what fully developed exosome–nanomaterial theranostics may look like. Hill et al. engineered exosome-coated Prussian blue nanoparticles and demonstrated BBB crossing, photoacoustic imaging detectability in orthotopic glioblastoma, and localized photothermal treatment under near-infrared (NIR) irradiation (Hill et al., 2024). Liu et al. extended this concept by constructing second near-infrared window (NIR-II)-fluorescent exosome–liposome hybrid vehicles for subcutaneous glioblastoma, showing that the hybrid platform enabled NIR-II fluorescence imaging-guided and targeted NIR-II photothermal therapy while maintaining colloidal stability and biocompatibility (Liu et al., 2025). Although neither study was performed in ischemic stroke, both are directly relevant to stroke-oriented hybrid design because they demonstrate that exosome–nanomaterial hybrids can unify brain lesion localization, imaging readout, and externally triggered treatment in a single platform (Hill et al., 2024; Liu et al., 2025).

Stimulus-responsive activation has likewise been demonstrated more clearly in adjacent CNS disorders than in stroke itself. Jiang et al. reported ROS-responsive biomimetic exosome–liposome hybrid nanovesicles for Alzheimer’s disease, in which the hybrid codelivered siBACE1 and pTREM2, penetrated the BBB, and enhanced lesion accumulation through a combination of exosome homing and angiopep-2 assistance (Jiang et al., 2024). In traumatic brain injury, Li et al. developed a ROS-responsive nanosystem camouflaged by hybrid membranes of platelets and engineered extracellular vesicles, showing lesion-oriented delivery and oxidative-stress-triggered cargo release in the injured brain (Li Y. et al., 2024). Han et al. further advanced this engineering logic in glioblastoma by designing an exosome-membrane-disguised thermoresponsive delivery system capable of BBB penetration and controlled drug release (Han et al., 2025). Physical stimulation has also begun to converge with exosome delivery in neurodegeneration: Yan et al. recently showed that low-intensity ultrasound enhanced exosome uptake and, when combined with intranasal human adipose-derived stem cell-derived exosomes (hADSC-Exos), improved cognition and hippocampal neurogenesis in amyloid precursor protein/presenilin 1 (APP/PS1) mice without overt toxicity (Yan et al., 2026). These studies do not constitute direct stroke evidence, but they strongly suggest that oxidative stress, local heating, or ultrasound can be harnessed as controllable triggers once exosome-based carriers have achieved sufficient brain access.

**Figure 4:**
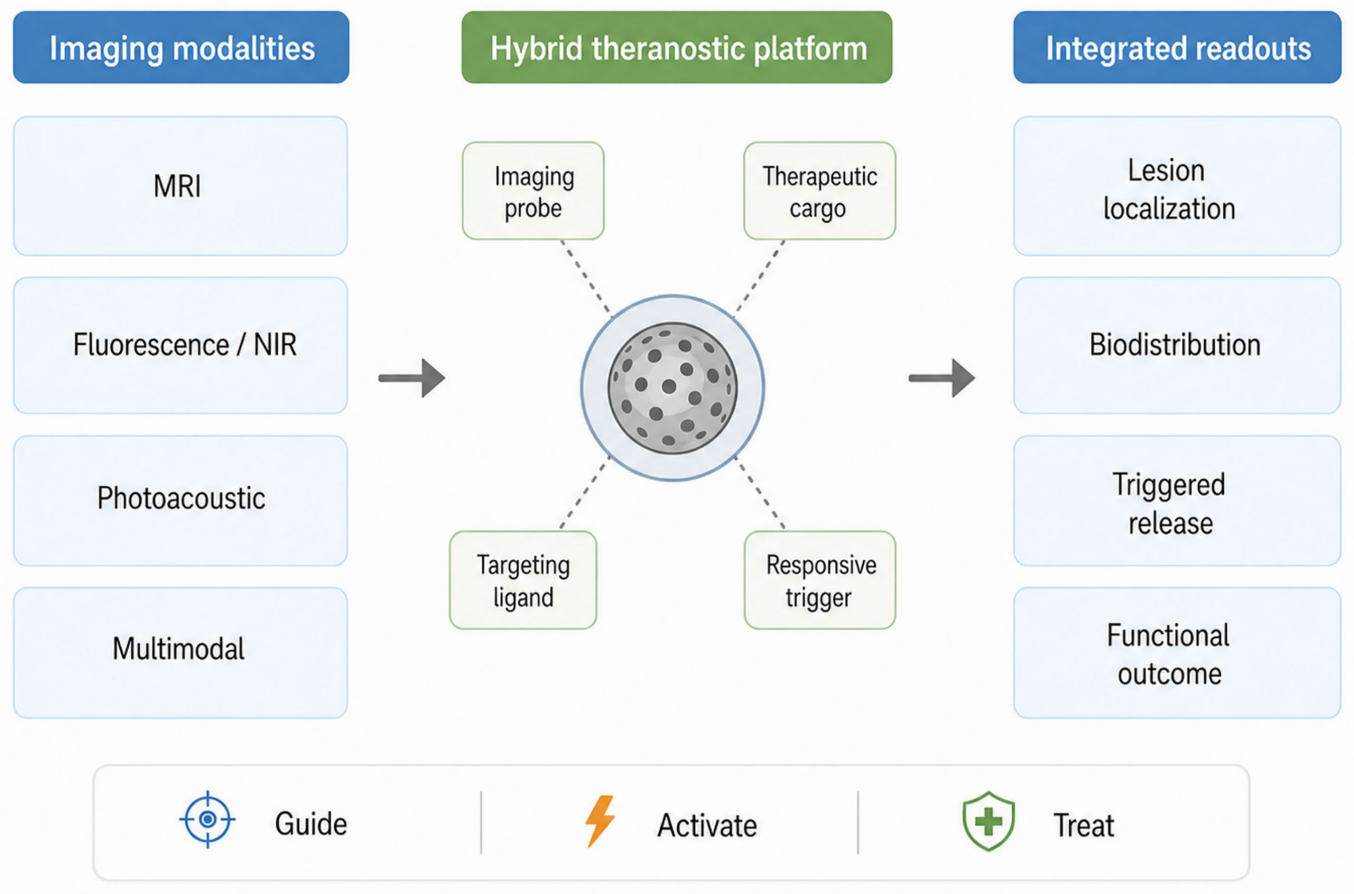
Imaging and theranostic integration. Theranostic systems can incorporate inorganic contrast agents, NIR or photoacoustic probes, magnetic modules, ultrasound-assisted delivery, and biomarker-responsive activation to link lesion visualization with therapeutic release.

Taken together, the current literature supports a staged interpretation of the field. In ischemic stroke, the most convincing evidence so far lies in MRI-trackable EVs, magnetic guidance, and focused-ultrasound-assisted lesion targeting (Hu et al., 2024; Helsper et al., 2024; Helsper et al., 2025; Alptekin et al., 2025). By contrast, the more elaborate theranostic architectures—photoacoustic imaging, NIR-II fluorescence guidance, ROS-triggered activation, and thermal-responsive release—have been demonstrated more fully in glioblastoma, Alzheimer’s disease, and traumatic brain injury models (Jiang et al., 2024; Hill et al., 2024; Liu et al., 2025; Li Y. et al., 2024; Han et al., 2025; Yan et al., 2026). For ischemic stroke, therefore, the next major advance will likely come from combining two lines of work, namely stroke-relevant lesion targeting and BBB penetration, and precisely activatable exosome–nanomaterial hybrid systems (Figure 4).

Theranostic hybrids are most useful when the imaging or activation module improves therapeutic decision-making or clarifies mechanism. MRI labels, magnetic modules, NIR probes, photoacoustic agents, and ultrasound-responsive components increase system complexity and may add safety burdens. Their inclusion is therefore best justified when they enable lesion confirmation, dose guidance, retention assessment, triggered release, or treatment-response monitoring.

### 4.4 Promotion of neurovascular repair and functional recovery

Beyond acute neuroprotection, one of the strongest rationales for exosome-based nanomedicine in ischemic stroke is its capacity to promote coordinated neurovascular repair and durable functional recovery. Recent work with mesenchymal-stem-cell-derived exosomes shows that these benefits are not mediated by a single pathway. Liu et al. reported that bone-marrow-mesenchymal-stem-cell-derived exosomes reduced infarct volume and improved neurological outcome while engaging astrocytic IL-33/ST2 signaling in ischemic models (Liu et al., 2023). Hu et al. further demonstrated that bone marrow MSC (BMSC)-derived exosomes promoted post-stroke angiogenesis through miR-21-5p, with associated increases in VEGF/VEGFR2 and Ang-1/Tie-2 signaling and higher microvessel density in ischemic tissue (Hu et al., 2022). In a complementary study, hiPSC-MSC-derived extracellular vesicles reduced infarct burden, improved motor behavior, and enhanced VEGF/CXCR4-associated angiogenesis in preclinical stroke models (Lu et al., 2023). Together, these studies indicate that exosome-mediated recovery after stroke is not limited to immediate cytoprotection, but includes active reconstruction of trophic, vascular, and peri-infarct repair programs.

Neural-lineage exosomes appear particularly relevant when the repair goal includes neurogenesis, neurite remodeling, and sustained structural recovery. Gu et al. showed that neural-stem-cell-derived exosomes delivered through an adhesive hydrogel promoted cerebral angiogenesis and improved neurological function in ischemic stroke (Gu et al., 2023). Zhang et al. later refined this sustained-delivery concept by integrating Buyang Huanwu decoction (BHD)-preconditioned neural stem cell (NSC)-derived exosomes into a hydroxypropyl methylcellulose (HPMC)-based nanohydrogel, which prolonged exosome retention and promoted angiogenesis, nerve regeneration, and behavioral recovery after MCAO (Zhang Q. et al., 2025). Engineered neural-stem-cell exosomes have also been used to strengthen reparative signaling. Zhu et al. loaded BDNF into human neural stem cell (hNSC)-derived exosomes and showed that the engineered vesicles inhibited microglial activation and promoted differentiation of endogenous NSCs into neurons in MCAO rats (Zhu et al., 2023). More recently, Zhao et al. reported that hNSC-Exos improved behavioral outcomes, reduced apoptosis, restored neurogenesis and neuroplasticity, and activated the PINK1/Parkin pathway to enhance mitophagy and mitochondrial repair (Zhao et al., 2025). These studies support the view that neural-lineage exosomes are particularly well suited for linking neurovascular remodeling with restoration of intrinsic neuronal plasticity.

Repair of the neurovascular unit also depends on endothelial and perivascular restoration rather than on neuronal rescue alone. Sun et al. showed that brain-microvascular-endothelial-cell-derived exosomes protected neurons from ischemia–reperfusion injury, promoted synaptic remodeling, improved regional cerebral blood flow, and enhanced motor behavior in MCAO/R mice (Sun et al., 2022). In a follow-up study, the same source class was shown to improve BBB integrity by increasing TEER, decreasing permeability, restoring tight-junction and basement-membrane proteins, and activating PDGF-PDGFRβ and Ang1/Ang2-Tie2 signaling, while also improving gait and rotarod performance after MCAO/R (Sun J. et al., 2025). These studies are important because they indicate that exosome-mediated recovery can be mediated through direct reinforcement of endothelial–pericyte communication and BBB structure, thereby creating a more permissive substrate for later neurorepair.

A fourth emerging theme is that exosome therapy can be potentiated by preconditioning, molecular enrichment, or combination with rehabilitation. Jiang et al. demonstrated that treadmill exercise synergized with bone-marrow-MSC-derived exosomes to further reduce infarct volume, improve neurological function, enhance synaptic formation, and promote axonal regeneration via c-Jun N-terminal kinase 1 (JNK1)/c-Jun-related signaling (Jiang et al., 2023). Wei et al. reported that Zeb2/Axin2-enriched BMSC-derived exosomes improved spatial memory, neurogenesis, dendritic plasticity, synaptic remodeling, and axonal preservation more effectively than control BMSC exosomes in MCAO rats (Wei et al., 2022). Zhang et al. further showed that N-methyl-D-aspartate receptor (NMDAR)-inhibitor-preconditioned mesenchymal-stromal-cell-derived EVs enhanced post-stroke recovery by reducing brain damage, improving cognition and sensorimotor integration, and promoting cerebrovascular reconstruction (Zhang X. L. et al., 2025). These data suggest that the reparative effect of exosome platforms is not fixed, but can be amplified by donor-cell conditioning, cargo enrichment, or coupling with pro-rehabilitative interventions. Consistently, dual-functionalized extracellular vesicles have been reported to promote brain repair and remodeling after ischemic stroke, while hippocampal-derived extracellular vesicles were shown to synergistically deliver active adenosine to support hippocampus-targeted cognitive recovery after stroke (Shi J. et al., 2025; Zhang L. K. et al., 2024).

Taken together, the recent literature indicates that neurovascular repair and functional recovery after stroke are best viewed as multi-compartment outcomes requiring coordinated effects on angiogenesis, BBB reconstruction, synaptic remodeling, neuronal regeneration, and behavioral restoration (Liu et al., 2023; Hu et al., 2022; Lu et al., 2023; Gu et al., 2023; Zhao et al., 2025; Sun et al., 2022; Sun J. et al., 2025; Zhu et al., 2023; Zhang Q. et al., 2025; Jiang et al., 2023; Wei et al., 2022; Zhang X. L. et al., 2025). The most promising next step will likely be to integrate repair-oriented biological exosome sources with hybrid engineering strategies that prolong retention, enhance lesion selectivity, and sustain controlled release within the peri-infarct niche.

Recovery-phase platforms should emphasize retention, durability, and coordinated neurovascular repair rather than rapid burst release. Hydrogels, adhesive depots, preconditioned exosomes, and cargo-enriched vesicles are especially relevant when the intended outcome involves angiogenesis, neurogenesis, synaptic remodeling, BBB reconstruction, or long-term functional recovery.

### 4.5 Administration routes and practical delivery challenges

Administration route is a core design variable because it determines biodistribution, exposure time, lesion exposure, delivery efficiency, and safety liabilities. For acute ischemic stroke, intravenous administration remains the most clinically compatible route because it can be integrated into emergency workflows and repeated when necessary. However, systemic injection also exposes EVs or hybrid particles to rapid dilution, hepatic and splenic clearance, pulmonary trapping, and interactions with circulating immune, complement, platelet, and coagulation systems. These limitations are especially relevant for stroke patients receiving reperfusion therapy or antithrombotic medication. In a murine ischemic stroke model, mesenchymal stem cell-derived EVs accumulated in injured brain regions after thrombolysis and attenuated tissue plasminogen activator (tPA)-induced BBB disruption and hemorrhagic transformation, suggesting that intravenous EVs may be useful as reperfusion adjuvants but also need to be evaluated in combination with clinically used thrombolytic regimens (Qiu et al., 2022). This study is important because it links route selection to a real acute-care scenario rather than testing EV delivery in isolation.

Systemic delivery may be strengthened by external guidance or engineered navigation. Magnetic extracellular nanovesicles derived from iron-oxide-loaded mesenchymal stem cells increased localization to the ischemic lesion and improved therapeutic outcome after systemic administration in transient middle cerebral artery occlusion models (Kim et al., 2020). This finding supports the value of adding a physical targeting module when the main obstacle is poor lesion accumulation after intravenous injection. In post-stroke cognitive impairment, superparamagnetic iron oxide-loaded human mesenchymal stem cell-derived exosomes enhanced brain targeting, improved mitochondrial function, and promoted behavioral recovery under magnetic attraction, further supporting magnetic guidance as a route-enhancing strategy for brain delivery (Hu et al., 2024). Nevertheless, magnetic or inorganic modules increase formulation complexity and may introduce additional requirements for particle stability, metal-related toxicity assessment, imaging compatibility, and field-guidance reproducibility. Therefore, intravenous hybrid systems should not be evaluated only by brain accumulation; they should also be tested for hemocompatibility, coagulation effects, organ retention, and compatibility with thrombolysis or thrombectomy-related treatment windows.

Intranasal administration offers a non-invasive route that may partially bypass systemic clearance and improve brain access through olfactory and trigeminal pathways. Zhou et al. showed that intranasally delivered brain-derived neurotrophic factor-loaded small EVs improved cerebral ischemia outcomes, supporting the feasibility of nose-to-brain EV delivery for ischemic stroke therapy (Zhou et al., 2023). In addition, intranasal Xingnaojing biomimetic nanoparticles have been reported as a non-invasive brain-targeted strategy for ischemic stroke treatment, further supporting the translational relevance of nasal administration in stroke nanomedicine (Li N. et al., 2024). This route is attractive for repeated dosing, subacute treatment, or recovery-phase intervention, but it also has practical limitations. Dose reproducibility may be affected by nasal mucosal retention, formulation volume, mucociliary clearance, local inflammation, and inter-individual anatomical variation. For hybrid systems, these issues may be further complicated by particle size, surface charge, mucoadhesion, and the risk that surface functionalization designed for brain targeting may also alter nasal residence and clearance.

Local or depot-based delivery is more suitable for subacute or recovery-phase repair than for routine acute treatment. Neural stem cell-derived exosomes loaded into adhesive hydrogel promoted cerebral angiogenesis and neurological recovery after ischemic stroke, indicating that hydrogel-based retention can extend exosome exposure within the injured tissue environment (Gu et al., 2023). Similarly, nanohydrogel-based delivery of preconditioned neural stem cell-derived exosomes prolonged local retention and promoted angiogenesis, nerve regeneration, and behavioral recovery in middle cerebral artery occlusion models (Zhang Q. et al., 2025). These studies suggest that local retention strategies are particularly useful when the therapeutic goal is sustained neurovascular repair rather than rapid acute neuroprotection. However, intracerebral, intraventricular, or hydrogel-based administration is invasive and may be difficult to integrate into standard acute stroke workflows unless combined with neurosurgical access, post-stroke repair procedures, or carefully selected subacute indications.

Physical delivery assistance provides another route-dependent strategy. Low-intensity pulsed focused ultrasound increased the delivery of endothelial progenitor cell-derived exosomes to ischemic regions and enhanced stroke recovery without evidence of excessive albumin leakage or CD45-positive cell accumulation in the reported model (Alptekin et al., 2025). This work is relevant because it shows that regional delivery can be improved without simply relying on uncontrolled BBB disruption. However, ultrasound-assisted delivery requires careful control of acoustic parameters, timing, microvascular status, and safety endpoints, because excessive barrier opening may increase edema, hemorrhagic risk, or inflammatory cell entry. For hybrid systems carrying inorganic contrast agents, magnetic components, or stimulus-responsive materials, physical guidance should therefore be justified by a measurable gain in lesion localization, dose reduction, release control, or treatment monitoring.

**Figure 5:**
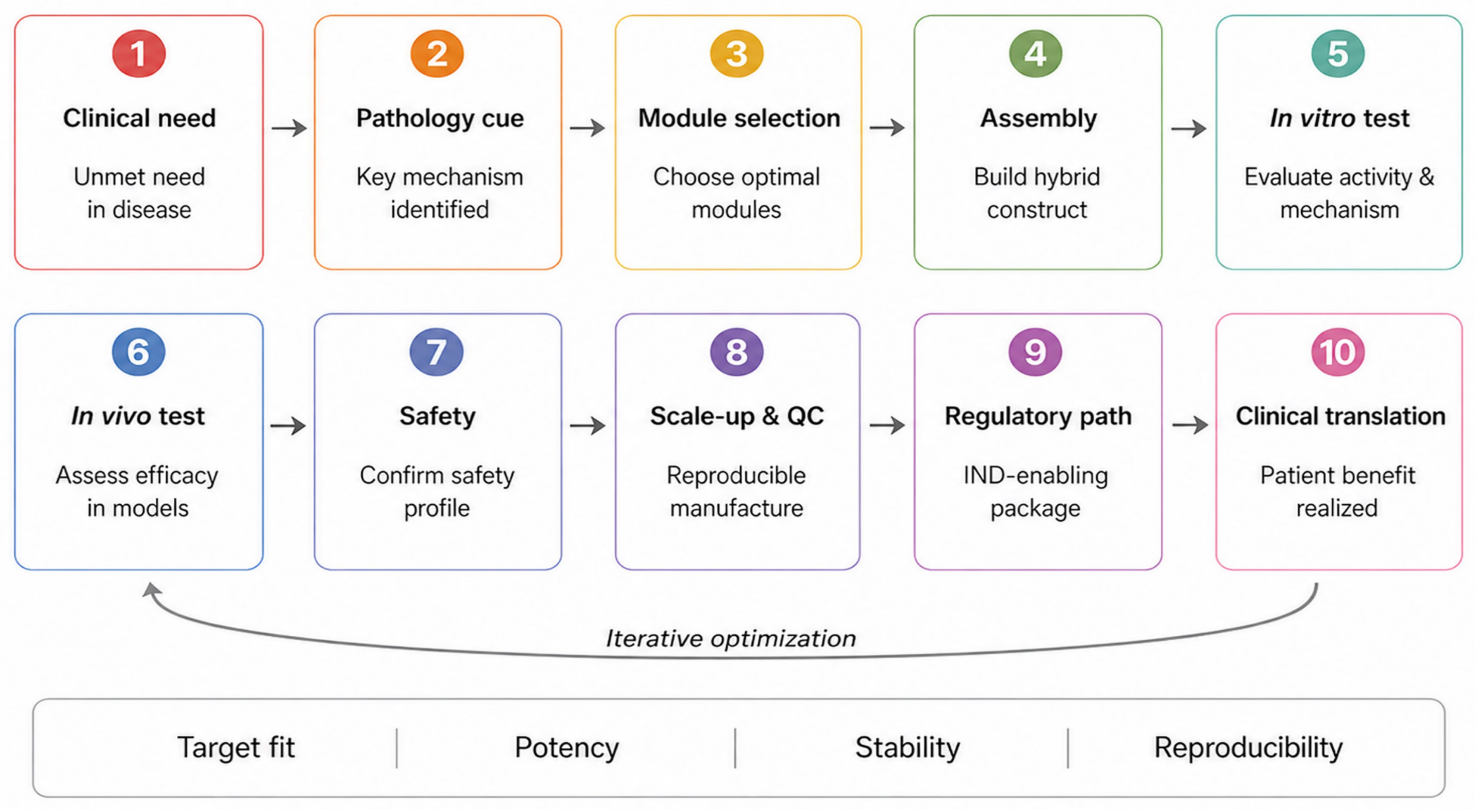
From pathology to product: a rational design roadmap. The proposed roadmap begins with clinical stage and pathological cue identification, followed by module selection, administration-route selection, hybrid assembly, functional validation, safety assessment, manufacturing scale-up, and regulatory positioning.

Overall, administration route should be selected according to the clinical stage and intended mechanism rather than treated as a technical afterthought. Intravenous or intra-arterial strategies are most rational for acute vascular-facing or reperfusion-adjacent interventions; intranasal delivery is attractive for non-invasive and repeated brain access; physical guidance may improve regional targeting when systemic delivery is insufficient; and local hydrogel or depot systems are more appropriate for sustained neurovascular repair. These route-dependent trade-offs are integrated into the pathology-to-product roadmap shown in Figure 5.

## 5 Discussion and translational roadmap

The revised design framework shifts the discussion from whether exosome-nanomaterial hybrids can work in experimental IS to what evidence is required before they can become credible translational products. The key challenge is to align biological function, engineering complexity, safety, manufacturability, and regulatory classification within one development pathway. This issue is particularly important for stroke because the same carrier property that improves lesion access, such as BBB interaction, immune-cell engagement, magnetic responsiveness, or prolonged tissue retention, may also introduce safety or quality-control liabilities if it is not evaluated with mechanism-matched assays (Qiu et al., 2022; Hu et al., 2024; Alptekin et al., 2025).

### 5.1 Potential side effects and safety liabilities

Potential adverse effects arise from three interacting sources: the exosomal component, the nanomaterial component, and the hybrid architecture itself. The exosomal component may carry unwanted donor-cell signals, inflammatory proteins, procoagulant membrane features, residual contaminants, or disease-state-dependent cargo. This concern is not theoretical: macrophage-derived exosomes enriched in THBS1 aggravated cerebral ischemia–reperfusion injury by inducing endothelial ferroptosis and BBB disruption, showing that exosomal cargo can actively worsen vascular injury under pathological conditions (Liu et al., 2025). Similarly, exosomes released from hypoxic vascular smooth muscle cells transferred Src and promoted M1 microglial polarization, thereby exacerbating ischemic brain injury (Zhang X. et al., 2024). These studies indicate that exosome source and donor-cell state must be treated as safety-critical variables rather than as interchangeable production details.

The nanomaterial component may introduce additional risks, including complement activation, oxidative stress, metal or polymer persistence, organ accumulation, dose-dependent endothelial toxicity, and interference with coagulation or immune pathways. Magnetic extracellular nanovesicles improved ischemic-lesion targeting after systemic administration, but this benefit depended on iron-oxide-associated engineering and external magnetic guidance, meaning that metal retention, magnetic-field reproducibility, and long-term biodistribution should be evaluated alongside therapeutic efficacy (Kim et al., 2020). In post-stroke cognitive impairment, superparamagnetic iron oxide-loaded MSC exosomes enhanced brain targeting and behavioral recovery, but this type of platform similarly requires safety assessment of the inorganic module in addition to conventional exosome characterization (Hu et al., 2024).

The hybrid construct may further create risks that are not present in either component alone. Hybridization can alter biodistribution, prolong retention, change cellular uptake, modify endosomal escape, and increase interaction with the BBB or circulating blood components. In the acute stroke setting, these concerns are amplified because patients may receive tissue plasminogen activator (tPA), antiplatelet agents, anticoagulants, or thrombectomy. In a murine ischemic stroke model, MSC-derived extracellular vesicles attenuated tPA-induced BBB disruption and hemorrhagic transformation, suggesting that EV-based platforms may be useful as reperfusion adjuvants but should be tested specifically under thrombolysis-relevant conditions rather than only in isolated stroke models (Qiu et al., 2022). Focused-ultrasound-assisted delivery also illustrates this balance: low-intensity pulsed focused ultrasound increased exosome accumulation in ischemic regions without increasing albumin leakage or CD45-positive cell accumulation in the reported model, but this finding also implies that BBB opening, inflammatory-cell entry, and hemorrhagic risk should be measured explicitly whenever physical delivery assistance is used (Alptekin et al., 2025).

A minimum safety package should therefore include hemocompatibility, complement activation, cytokine release, platelet aggregation, coagulation parameters, endothelial toxicity, BBB integrity, organ biodistribution, repeated-dose tolerability, and long-term clearance. For BBB-directed systems, safety should not be inferred from brain accumulation alone. It should be demonstrated that the platform crosses or engages the BBB without worsening edema, hemorrhagic transformation, tight-junction loss, or neurovascular inflammation. Recent safety guidance for EV-based therapeutic products emphasizes that manufacturing, quality evaluation, non-clinical safety, and clinical development should be considered together rather than as separate downstream tasks (Takakura et al., 2024).

### 5.2 Functional heterogeneity and quality control

Heterogeneity should not be viewed only as an analytical inconvenience. For exosome-nanomaterial hybrids, heterogeneity is functional: different vesicle subpopulations may carry different membranes, cargoes, uptake preferences, immune effects, and hybridization efficiencies. This point is supported by single-particle studies showing that EVs and synthetic nanoparticles can produce platform-dependent differences in size, counting, and phenotyping readouts, indicating that one analytical method cannot define a hybrid formulation adequately (Arab et al., 2021). Single-vesicle and single-molecule analyses of engineered EVs further showed that bulk cargo measurements can mask large vesicle-to-vesicle differences in loading efficiency, supporting the need to define the active particle population rather than relying only on total protein or total particle number (Silva et al., 2021).

A practical quality-control framework should include identity, purity, potency, stability, and function. Identity requires EV markers and material-specific signals. Purity requires quantification of protein aggregates, free liposomes, free nanoparticles, residual reagents, nucleic-acid contaminants, and endotoxin. Potency should be linked to the intended mechanism, such as BBB transport, ROS scavenging, anti-NETosis activity, microglial reprogramming, pro-angiogenic signaling, or intracellular RNA release. For example, EV-LNP fusion generated hybrid extracellular vesicles with improved mRNA loading and functional delivery, but the key quality attribute was not loading alone; it was whether the hybrid particles acquired functional endosomal-escape and *in vivo* mRNA-delivery capacity (Wang et al., 2025). Similarly, spontaneous hybridization of EVs with nucleic-acid-preloaded non-lamellar lipid nanoparticles showed that cargo loading, accessibility of EV membrane proteins, and retained biological activity should be evaluated together rather than separately (Bader et al., 2025). MISEV2023 provides a baseline reporting standard, but hybrid formulations require additional evidence that the EV and nanomaterial components form a reproducible and functionally active delivery platform (Welsh et al., 2024).

### 5.3 Manufacturing, scalability, and regulatory positioning

Translational development will require moving from bespoke laboratory assembly to scalable, good manufacturing practice (GMP)-compatible production. Key bottlenecks include donor-cell source control, culture medium, culture geometry, isolation method, batch yield, hybridization reproducibility, sterilization or bioburden control, storage stability, release testing, and potency assays. Manufacturing conditions can change EV yield and biological function. Haraszti et al. showed that exosomes produced from three-dimensional MSC cultures by tangential flow filtration had higher yield and improved functional activity compared with conventional approaches, indicating that production format can affect both quantity and potency (Haraszti et al., 2018). Large-scale bioreactor production of MSC-derived EVs has also been used to evaluate critical quality attributes such as identity, sterility, potency, and therapeutic efficacy, supporting the feasibility of scalable production but also emphasizing the need for process-linked quality control (QC) (Kink et al., 2024).

Clinical-grade EV production requires more than scale-up of particle number. Pachler et al. developed a GMP-grade protocol for human MSC-derived EVs using an animal-component-free, EV-depleted platelet lysate-based medium, illustrating that culture supplements and upstream materials must be controlled to avoid contaminating or confounding EV products (Pachler et al., 2017). Mendt et al. reported bioreactor-based large-scale production of GMP-grade MSC-derived exosomes engineered with siRNA, including shelf-life, biodistribution, toxicology, and efficacy testing, illustrating how clinical-grade exosomes can link manufacturing, quality control, safety, and function (Mendt et al., 2018). More recently, a clinical-grade human adipose MSC-derived EV (haMSC-EV) study standardized quality criteria, assessed lot-to-lot consistency by RNA-seq and proteomics, evaluated storage stability, and performed repeated-dose preclinical toxicity testing, showing how QC and safety assessment can be integrated into product development rather than added at the end (Wang et al., 2024).

For exosome-nanomaterial hybrids, these manufacturing issues become more complex because the final product contains both biological and synthetic modules. The EV component requires donor-cell and culture-process control, whereas the nanomaterial component requires control of particle size, composition, surface chemistry, residual reagents, and stability. The hybridization step adds another critical process parameter because incomplete fusion or inconsistent co-assembly may generate a mixture of free EVs, free nanoparticles, and true hybrid particles. Therefore, release testing should not only measure EV identity and nanomaterial identity separately, but also quantify the co-assembled hybrid fraction and its mechanism-linked potency (Evers et al., 2022; Son et al., 2025; Wang et al., 2025; Bader et al., 2025).

Regulatory positioning is also unresolved. Depending on source, cargo, and material component, a hybrid may resemble a biologic, a cell-derived product, a gene-delivery vehicle, a nanomedicine, an imaging agent, or a drug-device combination. A purely material-based regulatory logic is inadequate because the exosomal component contributes biological identity and activity. Conversely, a purely biologic logic is incomplete when inorganic, polymeric, imaging, magnetic, or externally activated modules determine safety and function. For this reason, the most defensible translational strategy is to define the product by critical quality attributes and mechanism-linked potency rather than by nomenclature alone. Recent EV safety guidance and MISEV2023 reporting principles provide useful baselines, but hybrid products require additional release criteria for hybridization efficiency, synthetic-module retention, free-material contamination, and stimulus-responsive function (Takakura et al., 2024; Welsh et al., 2024).

### 5.4 Pre-competitive roadmap and grand challenges

Several pre-competitive initiatives would accelerate the field. First, a shared characterization dataset for hybrid EV-nanomaterial formulations should define minimal and advanced assays for size, composition, hybridization, cargo loading, potency, and stability. This dataset should include orthogonal single-particle methods because different platforms can produce different sizing, counting, and phenotyping outputs for EVs and synthetic nanoparticles (Arab et al., 2021). Second, standard BBB and IS-relevant validation panels should be established, including *in vitro* BBB models, organ biodistribution, thromboinflammatory assays, and recovery-phase functional readouts. Human-cell-based and three-dimensional neurovascular-unit models are particularly relevant for this validation stage because they better reflect endothelial, neuronal, glial, and pericyte interactions during ischemic injury (Pang et al., 2024). The cubosome-exosome study illustrates why such panels are necessary: hybrid formulation ratios affected BBB uptake and transport, indicating that delivery performance cannot be inferred from particle assembly alone (Son et al., 2025). Third, benchmark formulations should be compared across laboratories to separate true biological improvement from formulation-specific artifacts. Fourth, manufacturing and release-test templates should be developed early rather than after proof-of-concept efficacy, because culture format, purification strategy, and storage conditions can alter EV yield, purity, and potency (Haraszti et al., 2018; Wang et al., 2024).

Five grand challenges are proposed. First, the field should develop a single platform that demonstrates sequential cue-responsive release *in vivo* across acute and subacute IS stages, rather than showing only one trigger in one pathological window. Second, single-particle quality-control methods should be established to identify the active hybrid subpopulation and distinguish true hybrids from free EVs or free synthetic particles (Arab et al., 2021; Silva et al., 2021). Third, safety should be proven in comorbidity-relevant stroke models, including aged, diabetic, hypertensive, thrombolysed, anticoagulated, or thrombectomy-relevant animals, because acute stroke patients rarely resemble young healthy experimental animals (Qiu et al., 2022). Fourth, scalable GMP-compatible production workflows should preserve both EV identity and nanomaterial function while controlling donor-cell state, culture medium, hybridization efficiency, free-material contamination, and storage stability (Kink et al., 2024; Pachler et al., 2017; Mendt et al., 2018; Wang et al., 2024). Fifth, clinical translation studies should prospectively align administration route, therapeutic window, imaging readout, mechanism-matched biomarkers, and release specifications rather than selecting these elements retrospectively after efficacy has already been claimed.

## 6 Conclusion

Exosome-nanomaterial hybrids provide a promising but technically demanding framework for IS therapy. Their value lies not simply in combining natural vesicles with synthetic materials, but in using each module to answer a defined pathological requirement of the ischemic microenvironment. A microenvironment-informed, mechanism-driven, and translationally aware design strategy can help the field move from descriptive platform development toward rational therapeutic engineering. If future studies connect lesion-stage biology, modular design, functional validation, safety, quality control, and scalable manufacturing, exosome-nanomaterial hybrids may not only advance IS treatment but also reshape the broader paradigm of CNS drug delivery.
